# Inferring gene regulatory networks by hypergraph generative model

**DOI:** 10.1016/j.crmeth.2025.101026

**Published:** 2025-04-11

**Authors:** Guangxin Su, Hanchen Wang, Ying Zhang, Marc R. Wilkins, Pablo F. Canete, Di Yu, Yang Yang, Wenjie Zhang

**Affiliations:** 1 School of Computer Science and Engineering, The University of New South Wales, Sydney, NSW, Australia; 2 ARC Centre of Excellence for the Mathematical Analysis of Cellular Systems (MACSYS), Melbourne, VIC, Australia; 3 Australian Artificial Intelligence Institute, The University of Technology Sydney, Sydney, NSW, Australia; 4 School of Computer Science and Technology, Zhejiang Gongshang University, Zhejiang, China; 5 Systems Biology Initiative, School of Biotechnology and Biomolecular Sciences, The University of New South Wales, Sydney, NSW, Australia; 6 Frazer Institute, Faculty of Health, Medicine and Behaviour Sciences, The University of Queensland, Brisbane, QLD, Australia; 7 Ian Frazer Centre for Children’s Immunotherapy Research, Child Health Research Centre, Faculty of Health, Medicine and Behaviour Sciences, The University of Queensland, Brisbane, QLD, Australia

**Keywords:** gene regulatory networks, scRNA-seq, hypergraph representation learning

## Abstract

We present hypergraph variational autoencoder (HyperG-VAE), a Bayesian deep generative model that leverages hypergraph representation to model single-cell RNA sequencing (scRNA-seq) data. The model features a cell encoder with a structural equation model to account for cellular heterogeneity and construct gene regulatory networks (GRNs) alongside a gene encoder using hypergraph self-attention to identify gene modules. The synergistic optimization of encoders via a decoder improves GRN inference, single-cell clustering, and data visualization, as validated by benchmarks. HyperG-VAE effectively uncovers gene regulation patterns and demonstrates robustness in downstream analyses, as shown in B cell development data from bone marrow. Gene set enrichment analysis of overlapping genes in predicted GRNs confirms the gene encoder’s role in refining GRN inference. Offering an efficient solution for scRNA-seq analysis and GRN construction, HyperG-VAE also holds the potential for extending GRN modeling to temporal and multimodal single-cell omics.

## Highlights

• Hypergraph modeling enhances scRNA-seq representation by reducing sparsity
• Capturing cellular heterogeneity and gene modules uncovers complex GRN relationships
• HyperG-VAE surpasses benchmarks in predicting GRNs and identifying key regulators
• Tested on B cells, HyperG-VAE excels in gene regulation, clustering, and lineage tracing

## Motivation

Gene regulatory networks (GRNs) derived from single-cell RNA sequencing (scRNA-seq) data provide insights into the complex interactions between transcription factors (TFs) and target genes. This capability enables a detailed understanding of gene expression regulation and cellular function across diverse populations. However, addressing both cellular heterogeneity and gene modules remains a significant challenge, as current GRN inference methods struggle to bridge this gap. To address this limitation, we introduce hypergraph variational autoencoder (HyperG-VAE), an algorithm based on hypergraph representation learning that captures latent correlations among genes and cells, enhancing the imputation of contact maps.

## Graphical abstract

**Figure:**
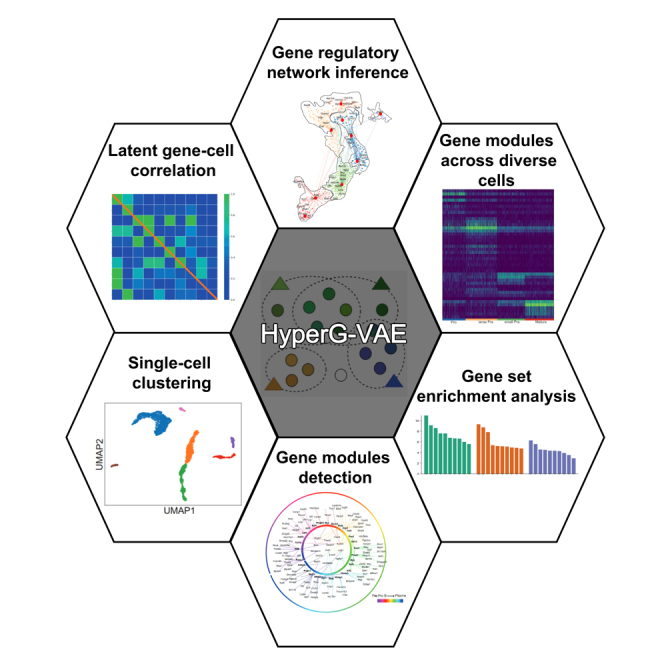

## Teaser

Su et al. present HyperG-VAE, a hypergraph-based model integrating cellular heterogeneity and gene modules for robust GRN inference from scRNA-seq data. By capturing latent gene-cell correlations, it overcomes sparsity, outperforms existing methods, and reveals key gene regulation patterns with robust downstream analysis.

## Introduction

Gene regulatory networks (GRNs) within single-cell RNA sequencing (scRNA-seq) datasets present a sophisticated interplay of transcription factors (TFs) and target genes, uniquely capturing the modulation of gene expression and thereby delineating the intricate cellular functions and responses within diverse cell populations.^1^ GRNs illuminate core biological processes and underpin applications from disease modeling to therapeutic design,^2^^,^^3^^,^^4^ empowering researchers to interpret the mechanisms of gene interactions within cells and leverage this understanding for medical and biotechnological innovations.^5^^,^^6^

Numerous methodologies have emerged for inferring GRNs from single-cell transcriptomic data. The algorithms emphasize co-expression networks in a statistical way (e.g., PPCOR^7^ and LocaTE^8^) or aim to decipher causal relationships between TFs and their target genes based on the analysis of the gene interactions among cells (e.g., DeepSEM^9^ and PIDC^10^). Despite their achievements, these algorithms still have inherent limitations. Specifically, these approaches mainly focus on cellular heterogeneity and overlook the critical importance of simultaneously considering cellular heterogeneity and gene module information in the model design. Generally, from the view of underlying principles, we can divide the methodologies into deep learning methods and traditional statistical algorithms. Many deep learning-based (e.g., DeepTFni^11^ and DeepSEM^9^) methodologies primarily build upon foundational models.^12^^,^^13^ The frequent oversight in these models is the inherent relationships between cells and genes, as informed by domain expertise. This often leads to models that compromise on explainability and narrow their application scope. For the traditional statistical algorithms, such as Bayesian networks^14^^,^^15^ and ensemble methods,^16^^,^^17^^,^^18^ it can be computationally expensive, and it remains a challenge to extend these methodologies to encompass broader nonlinear paradigms.

Additionally, the scRNA-seq data are frequently marred by noise and incompleteness, attributable to phenomena such as amplification biases inherent to reverse transcription and PCR amplification processes,^19^^,^^20^ as well as the issue of low quantities of nucleic acids in single cells. To get a more robust GRN, several methodologies^21^^,^^22^ leverage multi-omic datasets, capturing different kinds of cellular information to enrich the model’s comprehensiveness. However, integrating multi-omic datasets presents substantial challenges, particularly regarding harmonizing data from disparate sources and platforms, and could also introduce additional noise.^23^

To address the problems and construct a reliable GRN, we model scRNA-seq data as a hypergraph and present hypergraph variational autoencoder (HyperG-VAE), a Bayesian deep generative model to process the hypergraph data. Distinct from current approaches, HyperG-VAE simultaneously captures cellular heterogeneity and gene modules (in GRN analysis, gene modules refer to clusters of genes that are regulated together by the same set of TFs) through its cell and gene encoders individually during the GRN construction. Two encoders employ variational inference to learn stochastic representations of genes and cells, offering a more flexible and robust approach to managing real-world data complexities. This could be particularly effective in handling noise in scRNA-seq datasets, a capability that has been demonstrated in previous studies.^24^^,^^25^^,^^26^ Within a shared embedding space, the dual encoders of our model interact, boosting its cohesiveness. The joint optimization manner elucidates gene regulatory mechanisms within gene modules across various cell clusters, thereby augmenting the model’s ability to delineate complex gene regulatory interactions and significantly improving its explainability.

Our study evaluates the performance of HyperG-VAE in various scRNA-seq applications. These include (1) GRN inference, (2) cell embedding, (3) gene embedding, and (4) gene regulation hypergraph construction. Through benchmark comparisons, encompassing tasks like GRN inference, data visualization, and single-cell clustering, we establish that HyperG-VAE outperforms existing state-of-the-art methods. Additionally, HyperG-VAE demonstrates its utility in elucidating the regulatory patterns governing B cell development in bone marrow. Our model also excels in learning gene expression modules and cell clusters, which connect the gene encoder and cell encoder individually to boost gene regulatory hypergraph prediction. This integrated functionality of HyperG-VAE improves our comprehension of single-cell transcriptomic data, ultimately providing better insights into the realm of GRN inference.

## Results

### Framework overview

**Figure 1:**
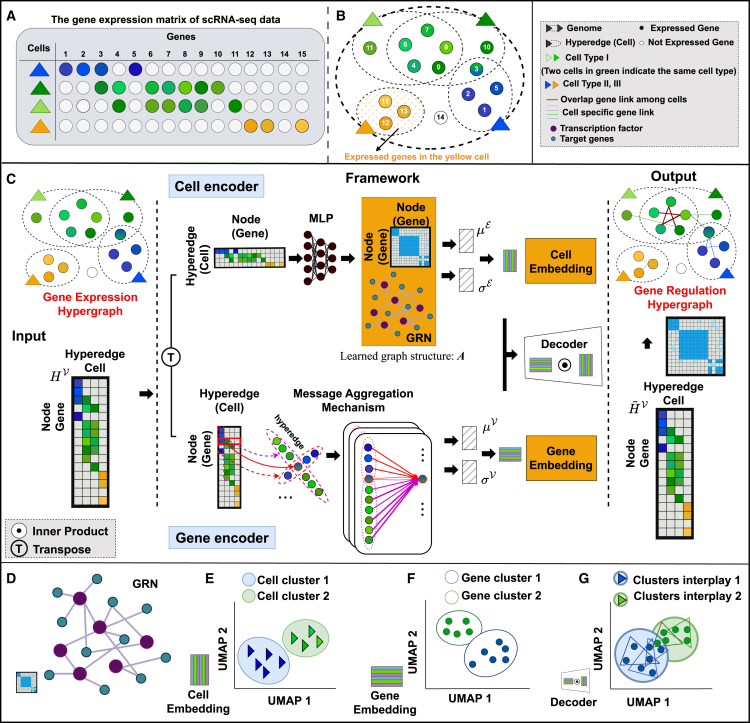
Overview of HyperG-VAE (A) HyperG-VAE, which takes the expression value matrix derived from scRNA-seq data as input. In the provided table, four cells exhibit expression across fifteen genes, with color gradients indicating varying gene expression levels (white circles mean no expression). (B) The colored circles with serial numbers denote distinct genes, expressed within specific cells, functioning as interlinked nodes. These nodes are interconnected by a singular hyperedge (small dashed ellipses) symbolizing the cell (triangle). Together, these nodes and hyperedges form a hypergraph structure. Node coloration reflects a composite of gene expression levels of the given gene across cells; for instance, gene 3 manifests a blend of green and blue hues. The largest dashed ellipse is the genome shared by all cells. (C) The neural network architecture of HyperG-VAE, where two encoders are designed to process the provided input matrix. The cell encoder uses the structural equation model (SEM) to discern cellular heterogeneity and form the GRN, while the gene encoder, employing a hypergraph self-attention mechanism, focuses on gene module analysis. The decoder subsequently reconstructs the input matrix, leveraging the shared latent space of both gene and cell embeddings. The inferred gene regulation hypergraph integrates cellular and gene representations, drawing on relationships derived from the learned GRN. (D–G) Downstream tasks that can be pursued by HyperG-VAE include GRN construction, clustering both cells and genes, and modeling the interplay between gene modules and cellular heterogeneity. Further details are provided in the legend, located in the top right corner.

We introduce HyperG-VAE, a Bayesian deep generative model specifically designed to address the complex challenge of gene regulation network inference using scRNA-seq data, which are represented as a hypergraph (Figure 1; STAR Methods). Our HyperG-VAE takes into account the interplay between gene modules and cellular heterogeneity, allowing for a more accurate representation of cell-specific regulatory mechanisms. This interplay could be incorporated into a hypergraph to capture the nuanced interactions of genes across diverse cellular states.

In the context of hypergraphs, we construct the hypergraph by representing cells as individual hyperedges, with the genes expressed in each cell serving as the nodes included within those hyperedges (Figures 1A and 1B). Specifically, let $H^{V}\in R^{m\times n}$ denote the scRNA-seq expression matrix, where *m* is the number of cells and *n* is the number of genes. The incidence matrix $M\in {\left\{0,1\right\}}^{m\times n}$ encodes the hypergraph structure: if a gene (node) *i* is expressed in cell (hyperedge) *j* ($H_{ij}^{V}>0$), then $M_{ij}=1$. This construction captures the relationship between cells and their expressed genes, enabling the sparse scRNA-seq data to be effectively represented as a hypergraph.

HyperG-VAE incorporates two encoders, a cell encoder and a gene encoder, enabling it to learn the hypergraph representation $H^{V}$ (Figure 1C) with structure M. The cell encoder generates cell representations $H^{E}$ in the form of hypergraph duality, facilitating the embedding of high-order relations via structural equation layers. GRN construction (Figure 1D) is realized in this structural equation layer through a learnable causal interaction matrix. In addition, the cell encoder can adeptly capture the gene regulation process in a cell-specific manner, elucidating a clearer landscape of cellular heterogeneity (Figure 1E). The gene encoder is specifically designed to process observed gene representations, denoted as $H^{V}$. Given that genes within a module generally manifest consistent expression profiles across cells, we employ a multi-head self-attention mechanism that is specifically designed for the hypergraph in this work. This not only discerns varying gene expression levels but also assigns appropriate weights to the genes expressed in the same cell during the message-passing phase. Thus, the gene encoder enhances the model’s ability to understand and integrate the intricate interdependencies among genes, thereby aiding in the effective embedding of gene clusters (Figure 1F). Finally, a hypergraph decoder is utilized to reconstruct the original topology of the hypergraph (Figure 1G) using the learned latent embedding of genes and cells. Utilizing the reconstructed hypergraph and the learned inter-gene relationships, we can also infer a gene regulatory hypergraph (Figure 1G). This hypergraph encompasses gene regulatory modules that span across various cell stages.

HyperG-VAE enhances GRN inference by incorporating the above two encoders to mutually augment each other’s embedding quality (Figure 1C) while preserving the high-order gene relations among cells, constrained by hypergraph variational evidence lower bound (STAR Methods). Specifically, the cell encoder incorporates a structure equation model (SEM) on gene co-expression space to infer the GRNs; the learning of gene modules by the gene encoder aids in the inference of GRNs since the gene module conceivably incorporates TF-target regulation patterns. By integrating the embedding of genes and cells through joint learning, we observe the substantial performance of downstream tasks (Figures 1D–1G), including the inference of GRNs, cell clustering, gene clustering, and interplay characterization between gene modules and cellular heterogeneity, among others.

### HyperG-VAE achieves accurate prediction of GRNs

We evaluate the performance on GRN inference of HyperG-VAE based on the setting of the BEELINE framework.^27^ Our evaluation encompassed seven scRNA-seq datasets. This includes two cell lines from humans and five mouse cell lines (more details can be found in the supplemental information). We evaluate GRN performance using two metrics: EPR, which assesses the enrichment of true positives among the top *K* predicted edges relative to random predictions, and AUPRC, which measures the area under the precision-recall curve to account for class imbalance. These metrics are applied across four types of ground-truth datasets: STRING,^28^ non-specific chromatin immunoprecipitation (ChIP)-seq,^29^^,^^30^^,^^31^ cell-type-specific ChIP-seq,^32^^,^^33^^,^^34^ and loss-/gain-of-function (LOF/GOF) networks.^34^ As recommended by Pratapa et al.,^27^ our analysis for each dataset prioritized the most variable TFs and the top *N* most-varying genes, where *N* is set to 500 and 1,000. We selected seven state-of-the-art baseline algorithms based on the evaluation of BEELINE to compare with HyperG-VAE: DeepSEM,^9^ GENIE3,^17^ PIDC,^10^ GRNBoost2,^18^ SCODE,^35^ ppcor,^7^ and SINCERITIES.^36^

**Figure 2:**
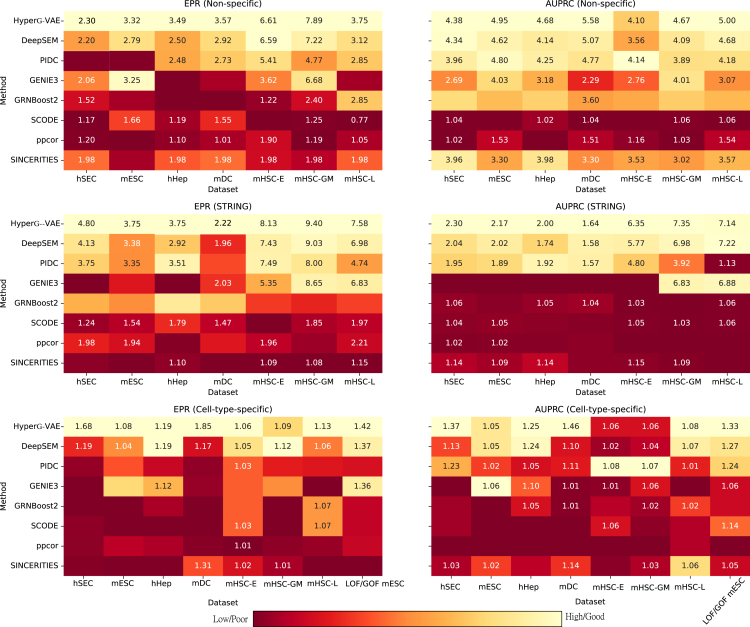
Benchmarks of different GRN inference methods on experimental scRNA-seq datasets by EPR and AUPRC scores The performance of HyperG-VAE is contrasted against seven alternative algorithms across seven datasets. Each dataset comprises all significantly varying transcription factors (TFs) and the 500 most-varying genes. These evaluations are based on four distinct ground-truth benchmarks: non-specific ChIP-seq, STRING, cell-type-specific ChIP-seq, and LOF/GOF. For each figure pair, the left image depicts the median EPR results, while the right image shows the median AUPRC outcomes. Results inferior to random predictions are excluded from the visualizations for clarity. The color scale in each dataset is normalized between 0 and 1 using a min-max scaling approach. EPR is defined as the odds ratio of true positives among the top K predicted edges, where K represents the number of edges in the ground-truth GRN, compared to random predictions. Similarly, the AUPRC ratio reflects the odds ratio of the AUPRC value between the model and random predictions.

Overall, HyperG-VAE demonstrates a discernible enhancement in performance when compared with other baseline methods in terms of both AUPRC and EPR metrics (Figures 2 and S2). For scaled results of datasets composed of all significantly varying TFs and the 500 most-varying genes (as shown in Figure 2), HyperG-VAE surpasses the seven other benchmarked methods in 40 of the 44 (91%) evaluated conditions. Compared with the second-best method (DeepSEM), HyperG-VAE enhances the results by at least 10% in 19 out of the 44 benchmarks. Furthermore, in comparison to other commendable approaches, such as PIDC and GENIE3, our approach registered significant enhancements. For PIDC, 38 out of 44 instances showed improvements of over 10%, with 27 surpassing 30% and 22 going beyond 50%. Similarly, with GENIE3, 33 out of 44 instances marked at least a 10% enhancement, 26 surpassed 30%, and an impressive 20 recorded at least a 50% increase. For results of datasets composed of all significantly varying TFs and the 1000 most-varying genes (Figure S2), HyperG-VAE achieves the best prediction performance on 84% (37/44) of the benchmarks. In comparison to the runner-up method, DeepSEM, HyperG-VAE outperforms by a margin of at least 10% in 17 of the 44 evaluated benchmarks. Notably, the average enhancement in EPR stands at 11.35%, while that in AUPRC is 7.16%.

With single-cell sequencing data, robustly inferring GRNs from limited cells is pivotal, especially for capturing rare cellular phenotypes and transient states.^9^^,^^11^ Here, we explore the fluctuations in EPR performance and the robustness of HyperG-VAE when confronted with limited training data (Figure S3A). We constructed mouse embryonic stem cell (mESC) datasets^37^ composed of all significantly varying TFs and the 500 and 1,000 most-varying genes, respectively, and evaluated the accuracy based on four unique ground-truth benchmarks by randomly subsampling single cells following the BEELINE benchmark.^27^ Upon adjusting the number of subsampled single cells to 400, 300, 200, 100, and 50, we registered average performance retentions of 94%, 92%, 91%, 80%, and 53%, respectively. Remarkably, when training with cell counts exceeding 100, a robust 79.17% (19/24) retained more than 90% of their performance, and for counts greater than 50, a compelling 87.50% (28/32) maintained above 80% efficacy. When utilizing cell-type-specific ChIP-seq as the benchmark, the performance remains notably stable, with an average performance retention of 93%. Furthermore, when assessed against the other three ground truths and the training cell count exceeds 50, there is only a modest decline in efficacy, averaging 88% performance retention in comparison to the median value derived from all cells. Beyond performance evaluation, we also examined HyperG-VAE’s scalability with expansive datasets (Figure S3B).

### HyperG-VAE reveals the gene regulation patterns of B cell development in bone marrow

**Figure 3:**
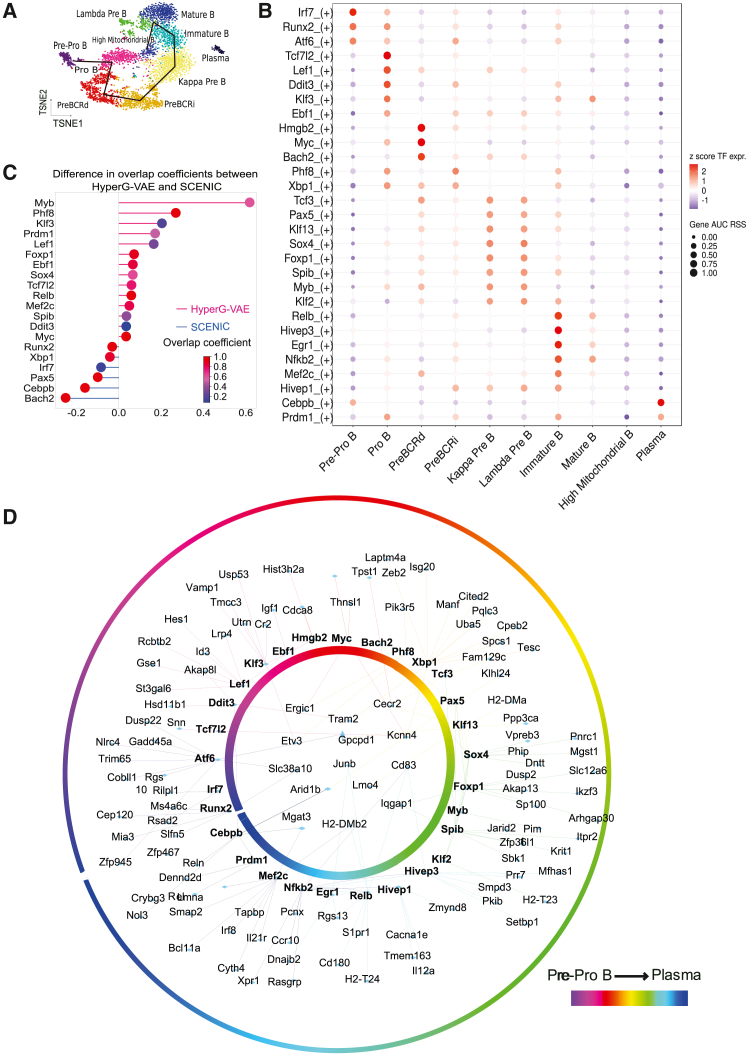
GRN prediction by HyperG-VAE across developmental B cell states in bone marrow (A) t-distributed stochastic neighbor embedding (t-SNE) visualization of cell embedding on the bone marrow B cell dataset; the embedding is learned by the cell encoder of HyperG-VAE. Black lines depict the trajectory from pre-pro-B cells to mature B cells. (B) Heatmap/dot plot showing TF expression of the regulon on a color scale and cell-type specificity (RSS [regulon specificity score]) of the regulon on a size scale. (C) The accuracy of GRN prediction by cross-validation with publicly available ChIP-seq datasets. The overlap coefficient quantifies the concordance between sets of target genes for each TF, as derived from GRN prediction and ChIP-seq database, respectively. The x axis represents the difference value of overlap coefficients between HyperG-VAE and SCENIC (default). Pink lines indicate superior performance by HyperG-VAE, while blue lines favor the default SCENIC. The dot plot illustrates the overlap coefficient of the more effective approach for each regulon, depicted on a color gradient. Cell states are arranged in a sequence that reflects the progression of bone marrow B cell development stages. (D) The GRN visualization for the bone marrow B cell dataset with ten states from pre-pro-B state to plasma state, as delineated by HyperG-VAE; the inner circle shows the co-binding of shared target genes, while the outer circle presents TF-focused target genes.

To evaluate HyperG-VAE’s proficiency in elucidating GRNs and to assess the effectiveness of both cell clustering embedding and gene module embedding components within HyperG-VAE, we deployed HyperG-VAE on scRNA-seq data of B cell development in bone marrow^38^ (more details of the data can be found in the STAR Methods and Table S3), as illustrated in Figure 3. The progression of B cell development from hematopoietic stem cells follows a sequential yet adaptable developmental pathway governed by interactions among environmental stimuli, signaling cascades, and transcriptional networks.^39^ Throughout this developmental trajectory, TFs play a pivotal role in regulating the cell cycle, differentiation, and advancement to subsequent developmental stages. These critical checkpoints encompass the initial commitment to lymphocytic progenitors, the specification of pre-B cells, progression through immature stages, entry into the peripheral B cell pool, B cell maturation, and subsequent differentiation into plasma cells.^40^ Each of these regulatory nodes is controlled by complex transcriptional networks, which, along with sensing and signaling systems, determine the final outcomes.

HyperG-VAE uncovers the cell embedding by dimensionality reduction and distinctly segregates the primary cell types across various stages of bone marrow B cell development (Figure 3A). Significantly, HyperG-VAE also effectively captures the linear progression of B cell development, spanning from early pro-B, late pro-B, large pre-B, small pre-B, and immature B to mature B cells. In our pursuit to unveil the gene regulation patterns in developmental B cells, our HyperG-VAE, in conjunction with SCENIC,^41^ successfully identifies established master regulators associated with different developmental stages (Figures 3B and S4), including pre-pro-B (Runx2), pro-B (Ebf1 and Lef1), large pre-B (Myc and Hmgb2), small pre-B (Tcf3 and Sox4), immature B (Relb and Egr1), mature B (Nfkb2), and plasma (Cebpb and Prdm1) cells.

Furthermore, we conducted a benchmark assessment to compare the performance of HyperG-VAE against SCENIC using its default settings. Using the ChIP-seq database,^33^ the accuracy was evaluated based on the degree of overlap coefficient between the ChIP-seq coverage and the predicted target genes from both methods. Our HyperG-VAE, when combined with SCENIC, demonstrates superior performance compared to the standard SCENIC approach, exhibiting higher accuracy in detecting TF-target patterns for the key TFs (as illustrated in Figure 3C). The comprehensive gene regulation network spanning the developmental B cells in the bone marrow is depicted in Figure 3D. We find that the GRNs show TF-target regulation patterns in two ways: TFs co-binding to shared predicted enhancers (the inner circle in Figure 3D) and TF-specific target genes (the outer circle in Figure 3D). We also observe that the cooperativity between TFs is stronger within cell types along the development path, indicating that some TFs are involved in multiple stages of B cell development.

### Gene expression module learning enhances HyperG-VAE in GRN inference

**Figure 4:**
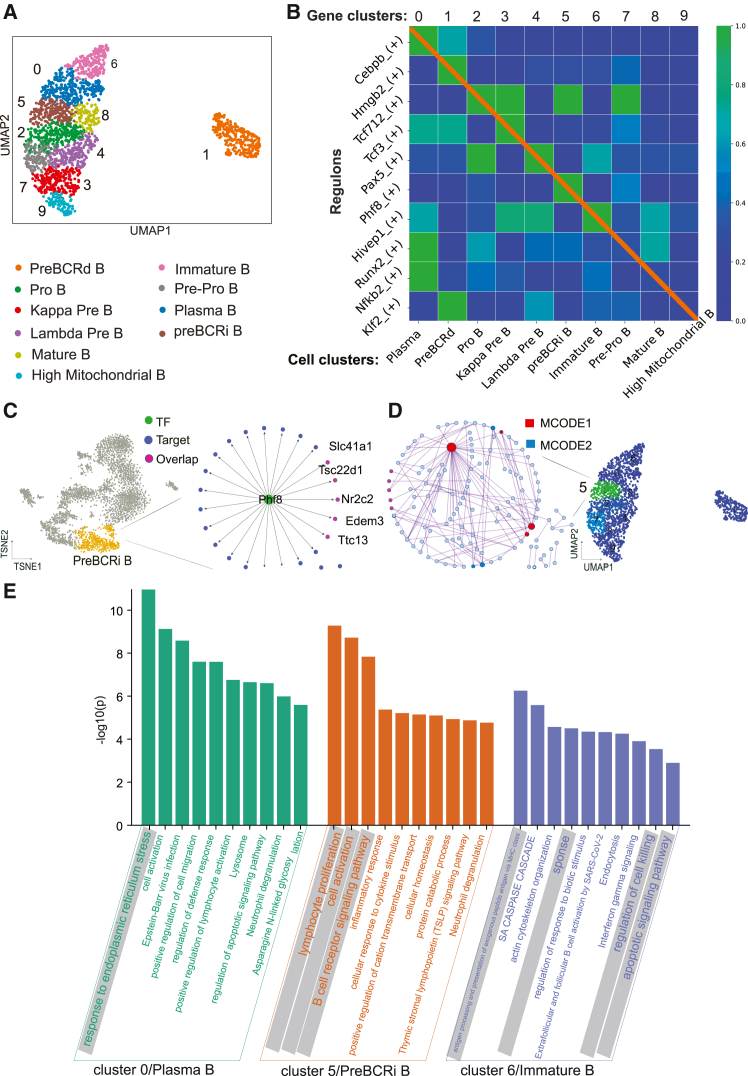
The interplay between gene embedding and cell clusters (A) Gene embedding by the gene encoder of HyperG-VAE on developmental B cell data. Gene clusters encoded by numbers are associated with different cell types by colors. (B) The heatmap illustrates normalized overlap values between gene clusters and TF regulons from different B cell states. Here, genes serve as a bridge to compute the overlap, with lighter colors representing larger overlap scores. (C) t-SNE visualization of cellular embeddings with highlighted pre-BCRi B cell state, together with the associated regulon, Phf8_(+), and related target genes. (D) Pathway enrichment analysis on gene cluster 5 with associated molecular complex detection (MCODE) network components. (E) Pathway enrichment analysis of different gene clusters. The shaded pathways show the dominant gene programs for each gene cluster.

Our HyperG-VAE model augments the GRN prediction by integrating gene space learning, as depicted in Figure 4C. HyperG-VAE uncovers the gene expression modules visualized by uniform manifold approximation and projection (UMAP)^42^ in Figure 4A. By associating these gene modules with the key TFs and corresponding target genes of pathways along B cell development, we annotate the gene modules with specific cell types, indicating that these gene clusters are activated in different stages of developmental B cells (Figures 4A and 4B).

We further apply gene set enrichment analysis (GSEA)^43^ (STAR Methods) to investigate the gene clusters (Figures 4E and S1; Tables S5 and S6). The pathways identified through GSEA validate the accuracy of our gene cluster annotations. For example, large pre-B cells (cluster pre-BCRi [B cell receptor independent] B) are associated with signals initiating diverse processes, which include proliferation and recombination of the light-chain gene^44^; the GSEA results show the related pathways: lymphocyte proliferation, cell activation, and B cell receptor signaling pathway. Immature B cells exhibit B cell central tolerance, which is governed by mechanisms such as receptor editing and apoptosis.^45^ The pathways identified in the corresponding gene clusters include antigen processing and presentation of exogenous peptide antigen, DNA damage response, regulation of cell killing, and apoptotic signaling pathways. Plasma cells are terminally differentiated B lymphocytes that secrete immunoglobulins, also known as antibodies.^46^ Considering the substantial demands placed on these cells for secretory biological processes, the pathways associated with the relevant gene cluster shed light on the cellular response to endoplasmic reticulum stress.

We show that the gene modules are associated with different biological pathways during B cell development in the bone marrow. These gene modules implicitly incorporate the gene regulation patterns, leading to different cell types. On the other hand, distinct cell types of B cell clustering are engaged in various immunological environments,^39^^,^^47^ resulting in different signaling pathways for B cell activation and fate decisions. We exemplify this joint relationship with an example involving B cells at the large pre-B stage, as shown in Figures 4C and 4D. This specific cell state (Figure 4C) is characterized by gene regulation patterns associated with cell proliferation, reflected by the regulon Phf8(+).^48^ The corresponding gene cluster (Figure 4D) is linked to a molecular complex detection (MCODE) network, which belongs to the lymphocyte proliferation pathway (Figure 4E) and shares target genes with the Phf8(+) regulon.

Therefore, our HyperG-VAE reciprocally integrates these two concepts, cell clustering and gene module detection, with the aim of revealing GRNs. Concretely, the cell embedding process groups together similar cells that share common pathways, while the gene modules aggregate genes exhibiting similar regulation patterns, thereby enhancing the accuracy of GRN computations.

### HyperG-VAE constructs the cell-type-specific GRN on B cell development in bone marrow

**Figure 5:**
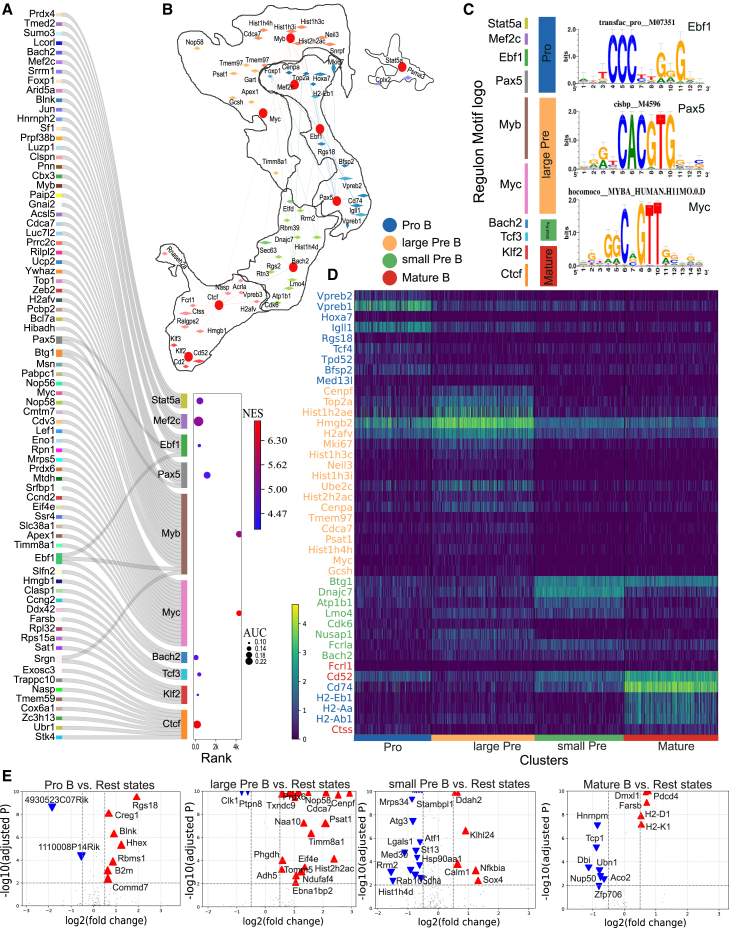
Cell-type-specific GRN analysis of developmental B cells in bone marrow (A) The Sankey diagram shows significant regulons and corresponding target genes of different states along B cell development in bone barrow, with the normalized enrichment score (NES) encoded by color shade and the area under the curve (AUC) score by dot size. (B) Gene regulatory hypergraph at the cell clustering level, illustrating the four principal B cell states as four hyperedges. Conserved TFs are highlighted with red dots, and target genes are depicted as diamonds, where size reflects the log fold change (logFC) in gene expression of a given state compared to others. Highly expressed genes are labeled in the figure. (C) The motif of significant TFs along the principal stages. Additional motif details can be found in Figure S5. (D) Heatmap displays the expression of the top genes, ranked by logFC, across cells classified into four distinct cell states. The genes are selected by the overlap of top logFC genes and predicted target genes. The genes’ color corresponds to the cell states in which the regulation pattern is predicted. (E) Volcano plot of differentially expressed genes of different states. The blue inverted triangles denote downregulated genes, and the red triangles denote upregulated genes.

We have demonstrated that gene modules associated with various biological pathways correspond to distinct cell types within bone marrow development in B cells. Essentially, these distinct gene regulation patterns influence cell fate commitment, leading to the development of diverse cell types with varying gene regulation profiles. Thus, we employ HyperG-VAE to investigate each individual state of developmental B cells and construct a more accurate GRN for B cells at specific developmental stages, as illustrated in Figure 5. B cell development in the bone marrow can be broadly categorized into four states: pro-B, large pre-B, small pre-B, and mature B.^40^ These four stages are visualized using UMAP, as depicted in Figure 5B. For each of these states, we employed HyperG-VAE to compute GRNs and uncover the predominant regulatory patterns, as illustrated in Figures 5A–5C. HyperG-VAE effectively reveals the key TFs and their associated target genes within each cell state. For example, in the pro-B state, Ebf1^49^ and Pax5^50^ play significant roles, while Myc^38^ stands out in the large pre-B state; Bach2^38^^,^^51^ is crucial in the small pre-B state, and Klf2^52^ and Ctcf^53^ are notable in the mature state.

The aforementioned TFs, along with their respective target genes, collectively constitute the regulons that characterize the four major cell states, allowing for the construction of a gene regulatory hypergraph at the cell clustering level (Figures 5A and 5B). For each major state, we overlap the top-predicted target genes by HyperG-VAE (Figure 5B) with the differentially expressed genes (DEGs; Figure 5E) and identify the principal marker genes (Figure 5D). Specifically, Ebf1 and Pax5 are essential in the pro-B state of bone marrow to maintain an early B cell phenotype characterized by the expression of B cell-specific genes such as Vpreb and Igll1 for surrogate light-chain production.^49^^,^^50^ In the large pre-B stage, the enriched regulons encompass the TF Myc^38^ and other genes related to the cell cycle, such as Mki67, Cenpf, Cenpa, and Hmgb2. Additionally, nucleosome-related genes, such as Hist1h2ae and Hist1h3c, are also enriched in this state due to the high rate of cell proliferation. In the small pre-B stage, both Bach2 and Btg1 restrain cell proliferation.^54^^,^^55^ It is noteworthy that the mature state markers H2-Ab1, H2-Eb1, H2-Aa, and Cd74 are assigned as target genes in the pro-B stage, suggesting that these genes may be actively repressed in the early B cell development stage.

### HyperG-VAE addresses cellular heterogeneity and learns the cell representation

Cellular heterogeneity is a hallmark of complex biological systems, manifesting as diverse cell types and states within scRNA-seq datasets.^56^ We hypothesize that the latent space inferred by the cell encoder of HyperG-VAE captures this biological variability among cells. Leveraging domain expertise, we can map these clusters to known cell types or states, ensuring that the computational predictions align with manual inspection and annotation. To evaluate the performance, we applied HyperG-VAE to three biologically relevant scRNA-seq datasets, including an Alzheimer’s disease (AD) dataset,^57^ a colorectal cancer dataset,^58^ and the widely used mouse brain dataset, known as the Zeisel dataset^59^ (more dataset details can be found in Table S4). To benchmark HyperG-VAE, we also compared its low-dimensional embeddings with those of six other algorithms: autoCell,^60^ DCA,^61^ scVI,^62^ DESC,^63^ SAUCIE,^64^ and scVAE.^65^ We followed the Louvain algorithm^66^ to cluster all the single cells into an identical number of clusters for each method (STAR Methods). To assess the precision of clustering against established reference labels, we employed four metrics: the adjusted Rand index (ARI), normalized mutual information (NMI), homogeneity (HOM), and completeness (COM). These metrics span a scale from 0, indicating random clustering, to 1, signifying perfect alignment with reference clusters, with superior values indicating enhanced accuracy.

**Figure 6:**
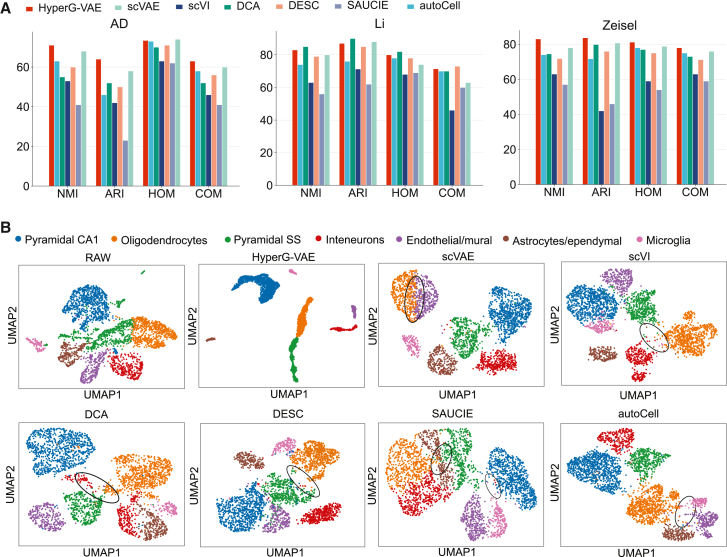
Benchmarks of single-cell clustering and embedding (A) The cell clustering performance of HyperG-VAE on the single-cell datasets compared with six baseline methods on four key metrics: NMI, ARI, COM, and HOM. NMI, normalized mutual information (the higher the value, the better); ARI, adjusted rand index (the higher the value, the better); COM, completeness (the higher the value, the better); HOM, homogeneity (the higher the value, the better). (B) UMAP visualization of latent representations on the Zeisel dataset for different methods. The black circles highlight areas with ambiguous classification boundaries.

Overall, the performance of HyperG-VAE surpasses that of its counterparts, as evidenced in Figure 6A. Specifically, for the Zeisel dataset, the clusters generated using HyperG-VAE align more closely with the existing cell type annotations, registering an NMI of 83.1% and an ARI of 83.7%. In comparison, the next best-performing algorithm, autoCell, recorded an NMI of 78.0% and an ARI of 80.6%. Furthermore, we evaluated HyperG-VAE’s latent space to determine its ability to capture the biological diversity among individual cells in the Zeisel dataset, as illustrated in Figure 6B. We visualized the data embedding by UMAP. In previous sections, we showed that HyperG-VAE effectively identifies gene expression differences within cells of the same type. The compact UMAP highlights distinct clusters while preserving intra-cluster heterogeneity, demonstrating its ability to capture both inter- and intra-cluster variability. Compared to other algorithms, the distinct separation observed with HyperG-VAE across most clusters indicates effective clustering, suggesting that HyperG-VAE’s cell encoder adeptly distinguishes between various cell states or types. While algorithms such as autoCell, scVI, and scVAE have achieved results that are comparable, the differentiation between their clusters is not as pronounced as with HyperG-VAE. For the remaining algorithms, the substantial overlap among clusters hinders the classifier from producing optimal results. Specifically, compared to other methodologies founded on conventional single-layer VAEs, the enhanced visualization capabilities of HyperG-VAE underscore the potential benefits of incorporating gene modules in cell embedding processes.

## Discussion

In this work, we introduce HyperG-VAE, a sophisticated model designed for the construction of GRNs. Uniquely, HyperG-VAE leverages a hypergraph framework, wherein genes expressed within individual cells are represented as nodes connected by distinct hyperedges, capturing the latent gene correlations among single cells. As a key algorithmic innovation of HyperG-VAE, the transformation of scRNA-seq data into a hypergraph offers unique advantages compared to existing GRN inference methods. These advantages include improved modeling of cellular heterogeneity, enhanced analysis of gene modules, increased sensitivity to gene correlations among cells, and improved visualization and interpretation of GRNs. This direct use of a hypergraph, as opposed to traditional pairwise methods like star expansion (SE) and clique expansion (CE),^67^ captures complex multi-dimensional relationships more effectively, avoiding the increased complexity and information loss associated with SE and CE. By maintaining the hypergraph’s original form, HyperG-VAE preserves the data’s full complexity and integrity, enhancing analytical depth and reducing computational demands.

In addition to modeling scRNA-seq data in a hypergraph, HyperG-VAE effectively integrates gene modules and cellular heterogeneity, demonstrating superior performance compared to existing methods. On the one hand, our study reveals that HyperG-VAE outperforms related existing state-of-the-art algorithms in GRN inference, cell type classification, and visualization tasks, respectively, as evidenced by its enhanced performance across several widely recognized benchmark datasets. On the other hand, we also utilize HyperG-VAE on scRNA-seq data of B cell development in bone marrow^38^ to evaluate its performance in a biologically relevant context. Firstly, HyperG-VAE achieves accurate prediction of GRNs and successfully identifies key master regulators and target genes across different developmental stages. Meanwhile, we cross-validated our results with publicly available ChIP-seq datasets,^33^ further demonstrating HyperG-VAE’s robust performance in predicting regulons based on GRN inference. Secondly, subsequent evaluations across various tasks further highlighted the effectiveness of HyperG-VAE’s carefully designed encoder components, with their synergistic interaction significantly bolstering the model’s overall performance. Specifically, the cell encoder within HyperG-VAE predicts the GRNs through a structural equation model while also pinpointing unique cell clusters and tracing the developmental lineage of B cells; the gene encoder uncovers gene modules that implicitly encapsulate patterns of gene regulation, thereby enhancing the accuracy of GRN predictions. To demonstrate this interaction, we highlight the shared genes between gene clusters and the predicted target genes within cell clusters. These shared genes are notably present in pathways identified by GSEA, signifying the connections between gene modules identified by gene encoders and cell clusters delineated by cell encoders.

Our proposed model, HyperG-VAE, holds promise as a foundational framework, adaptable to a multitude of biological contexts in future research endeavors. A promising future direction is extending HyperG-VAE into a heterogeneous hypergraph VAE by incorporating additional omics data, such as single-cell ChIP-seq. Integrating single-cell ChIP-seq data into HyperG-VAE would enhance GRN construction, complementing transcriptomic data and revealing upstream regulatory events. This kind of integration would enable seamless multi-omics data fusion and further advance GRN inference and cellular regulation analysis. Additionally, while the present model does not explicitly use metadata for genes and cells, future enhancements that integrate these metadata into the hypergraph-centric framework could significantly improve the representations of nodes (genes) and hyperedges (cells). The weights assigned to these hyperedges can also be factored into the model’s learning phase, offering a more comprehensive analysis. In the generative phase of HyperG-VAE, gene-cell interactions proceed through a cohesive mechanism, facilitating the development of a robust GRN underscored by the interplay between gene modules and cell clusters. Moreover, advancing to a single-cell-level, fine-grained gene-coexpression hypergraph study could further enhance our understanding of single-cell dataset analysis. Furthermore, subsequent research could explore the dynamic construction of temporal GRNs on chronological single-cell data, drawing upon the foundational principle of simultaneously considering cellular heterogeneity and gene modules, as demonstrated in this work.

Overall, HyperG-VAE provides a competitive solution for GRN construction and related downstream works. By combining cell-specific GRN inference, hypergraph-based gene module identification, and integrated cell-gene latent representations, HyperG-VAE delivers biologically relevant insights that extend beyond traditional GRN methods. It provides researchers with powerful tools to explore cell-specific regulatory mechanisms, identify gene modules, and generate testable predictions, thereby advancing our understanding of complex biological systems.

### Limitations of the study

Our HyperG-VAE leveraging the self-attention mechanism has undeniably propelled models to achieve remarkable performance.^68^^,^^69^^,^^70^^,^^71^ However, despite its prowess, self-attention-based models still have inherent limitations. Specifically, the self-attention’s quadratic complexity concerning sequence length presents challenges. For sequences of length *N*, it necessitates $O\left(N^{2}\right)$ computations, rendering it computationally demanding and memory inefficient, especially for longer sequences. Future efforts to address this limitation will explore adapting the techniques of attention matrix sparse factorization and positive orthogonal random features, as demonstrated in studies,^72^^,^^73^ to ease computational demands.

## Resource availability

### Lead contact

Requests for resources of this article and any additional information should be directed to the lead contact, Wenjie Zhang (wenjie.zhang@unsw.edu.au).

### Materials availability

This study did not generate new unique reagents.

### Data and code availability

• All datasets used in the work have been summarized in the key resources table.
• All original code has been deposited at https://github.com/guangxinsuuu/HyperG-VAE and is publicly available at https://doi.org/10.5281/zenodo.15028720 as of the date of publication.
• Any additional information required to reanalyze the data reported in this paper is available from the lead contact upon request.

## Acknowledgments

This work was supported by the ARC Centre of Excellence for the Mathematical Analysis of Cellular Systems (CE230100001), the Australian National Health and Medical Research Council (NHMRC) grant GNT2009554, and Children’s Hospital Foundation philanthropic funding.

## Author contributions

G.S., Y.Y., and H.W. designed the experiments. G.S., H.W., W.Z., Y.Y., and P.F.C. performed the experiments and analyzed the data. G.S., Y.Y., Y.Z., D.Y., M.R.W., and W.Z. performed the statistical analysis. D.Y., M.R.W., and W.Z. provided conceptual guidance. W.Z. and Y.Y. supervised the study. G.S., H.W., Y.Y., M.R.W., and W.Z. wrote the manuscript and prepared figures and tables. All authors reviewed and edited the manuscript.

## Declaration of interests

The authors declare no competing interests.

## STAR★Methods

### Key resources table

**Table:** 

REAGENT or RESOURCE	SOURCE	IDENTIFIER
**Deposited data**		

STRING	STRING^28^	https://string-db.org/
Cell-type Non-specific ChIP-seq	TRRUST^74^	https://www.grnpedia.org/trrust/
Cell-type Non-specific ChIP-seq	RegNetwork^30^	https://www.regnetworkweb.org
Cell-type Non-specific ChIP-seq	DoRothEA^29^	https://saezlab.github.io/dorothea/index.html
Cell-type specific ChIP-seq	ChIP-Atlas^33^	https://chip-atlas.org/peak_browser
Cell-type specific ChIP-seq	ESCAPE^34^	http//www.maayanlab.net/ESCAPE/download.php
Cell-type specific ChIP-seq	ChEA^75^	https://maayanlab.cloud/Harmonizome/dataset/CHEA+Transcription+Factor+Targets
lof/gof	ESCAPE^34^	http//www.maayanlab.net/ESCAPE/download.php
scATAC-seq	Fang et al.^76^	https://www.ncbi.nlm.nih.gov/geo/query/acc.cgi?acc=GSE126724
snmC-seq (DMR)	Luo et al.^77^	https://www.ncbi.nlm.nih.gov/geo/query/acc.cgi?acc=GSEGSE97179 Supplmentary Tables in Luo et al.
hHEP dataset^78^	GSE81252	https://www.ncbi.nlm.nih.gov/geo/query/acc.cgi?acc=GSE81252
hESC dataset^79^	GSE75748	https://www.ncbi.nlm.nih.gov/geo/query/acc.cgi?acc=GSE75748
mESC dataset^37^	GSE98664	http://www.example.com
mDC dataset^80^	GSE48968	https://www.ncbi.nlm.nih.gov/geo/query/acc.cgi?acc=GSE48968
mHSC dataset	GSE81682	https://www.ncbi.nlm.nih.gov/geo/query/acc.cgi?acc=GSE81682

**Software and algorithms**		

HyperG-VAE	This paper	https://doi.org/10.5281/zenodo.15028720
SCENIC	Aibar et al.^41^	https://github.com/aertslab/pySCENIC
Chip-Atlas	Oki et al.^33^	https://chip-atlas.org/target_genes
SCANPY	Wolf et al.^81^	https://scanpy.readthedocs.io/en/stable/
Metascape	Zhou et al.^43^	https://metascape.org/gp/index.html#/main/step1

### Method details

#### Preliminaries

##### Notation

Given a hypergraph G={V,E}, where $V=\left\{v_{1},\ldots ,v_{m}\right\}$ denotes the set of nodes, and $E=\left\{e_{1},\ldots ,e_{n}\right\}$ is the set of hyperedges. Within the hypergraph framework, it is possible for numerous nodes to be interconnected by a solitary hyperedge. Aligning the hypergraph framework with the gene regulation networks (GRNs) paradigm, the expressed genes are mapped as nodes while individual cells stand in as the hyperedges, thus crafting a representation of the cellular architecture as a hypergraph. We aim to approximate the real-world regulatory network $\tilde{A}$ by learning a causal interaction matrix A through HyperG-VAE. Both $\tilde{A}$ and A are square matrices, with their elements representing the levels of regulatory interaction between pairs of genes. In the context of hypergraphs, let $H^{V}\in R^{m\times n}$ represent the expression matrix of scRNA-seq dataset, where *m* represents the number of cells and *n* indicates the number of genes. and M signify the m×n incidence matrix. The matrix M is also of size m×n. If node *i* is linked to hyperedge *j* (gene *i* expressed in cell *j*), then $H_{ij}^{V}>0$ and $M_{ij}=1$. In the absence of such a link, both $H_{ij}^{V}$ and $M_{ij}$ are set to 0. For the hypergraph G, its dual is defined as $\overset{´}{G}=\left\{\overset{´}{V},\overset{´}{E}\right\}$. Here, V=E and $\overset{´}{E}$ comprises sets ${\overset{´}{e}}_{i}$ where each ${\overset{´}{e}}_{i}$ corresponds to edges in E that contain node $v_{i}$. As a direct consequence, the feature matrix of the dual, $H^{E}\in R^{n\times m}$, is the transpose of the feature matrix $H^{V}\in R^{m\times n}$ of G.

##### Structural Equation Model

Within the dual of scRNA-seq expression matrix $H^{E}$, we employ the Structural Equation Model (SEM),^74^ a statistical approach that integrates factor analysis and multiple regression, to model causal relationships among genes and deduce the intricate dynamics present within gene regulatory networks (GRNs), considering both observed and latent gene interactions. Specifically, our approach is rooted in the Linear SEM:

(Equation 1) $$H^{E}={\tilde{A}}^{T}H^{E}+Z\text{,}$$

Here, $Z\in R^{m\times d}$ is the intrinsic noise component following a Gaussian distribution denoted by N(0,I). The adjacent matrix $\tilde{A}$ indicates the conditional dependencies among genes. This characteristic implies a mechanism to derive $H^{E}$ from the noise matrix Z, expressed as:

(Equation 2) $$H^{E}={\left(I-{\tilde{A}}^{T}\right)}^{-1}Z\text{,}$$

This expression elucidates the relationship between $H^{E}$ and Z while highlighting the underlying network structure of the GRN as captured by the matrix $\tilde{A}$. In scRNA-seq, the data matrix (where rows represent cells and columns represent genes) captures complex gene dependencies. SEM models these dependencies by learning a matrix $\tilde{A}$ that represents conditional gene-gene relationships, while accounting for intrinsic noise through a Gaussian distribution. This enables HyperG-VAE to reconstruct GRNs and ensures biologically meaningful representations that reflect both direct and indirect gene interactions.

#### Hypergraph variational evidence lower bound

The input scRNA-seq expression matrix $H^{V}$ is often noisy and incomplete due to factors like amplification biases during reverse transcription and PCR amplification,^19^^,^^20^^,^^75^ can compromise the efficacy of basic autoencoders. These autoencoders risk overfitting to training data by solely penalizing reconstruction error, which are influenced by suboptimal expression matrices.^76^ To relief the problem, within HyperG-VAE, the hypergraph’s stochastic distribution is tailored to emphasize the latent spaces of nodes and hyperedges, rather than merely relying on observed inputs. Specifically, the node and hyperedge latent spaces are independently derived using distinct encoders and are subsequently refined according to Equation 3:

(Equation 3) $$\begin{array}{cc} L\left(H^{V};\theta ,\lambda \right) & =E_{q}\left[\log\;p\left(H^{V}|Z^{V},Z^{E};\lambda^{V}\right)\right]\\ & -\alpha KL\left(q\left(Z^{V}|H^{V};\theta^{V}\right)∥p\left(Z^{V}\right)\right)\\ & -\beta KL\left(q\left(Z^{E}|H^{E};\theta^{E}\right)∥p\left(Z^{E}\right)\right)\text{,} \end{array}$$

As a crucial loss function of HyperG-VAE, the Evidence Lower Bound (ELBO) is formulated with respect to the observed hypergraph node matrix $H^{V}$ and the parameters θ and λ which need to be estimated. Specifically, the expectation term, $E_{q}$, is the likelihood of the model’s reconstruction of the node matrix using the latent representations for nodes $Z^{V}$ and hyperedges $Z^{E}$. Moreover, the Kullback-Leibler (KL) divergence assesses the deviation of the learned latent distribution, $q\left(Z^{\cdot }|H^{\cdot }\right)$, from a designated prior $p\left(Z^{\cdot }\right)$. The coefficients *α* and *β* modulate the magnitude of this regularization.

#### HyperG-VAE node encoder

For the expression matrix $H^{V}$, each row $h_{i}$ delineates the expression profile of a gene across diverse cells. Concurrently, a particular gene might manifest across numerous cells and associate with other genes via distinct hyperedges $e_{k}$.

In the message-passing phase, row weights should account for expression coherence: genes within the same module typically exhibit consistent expression profiles across cells,^77^^,^^78^ warranting higher weights than genes with more variable expressions.

Based on the basic idea of GAT,^79^ we have devised an attention computation mechanism tailored for hypergraph, which enables (implicitly) specifying different weights to different nodes share a common hyperedge $e_{k}$. Multi-head attention was selected for the gene encoder to model the complex and dynamic relationships among genes. This mechanism enables the encoder to learn context-specific expression differences, allowing for the identification of reliable gene modules across diverse cells. By dynamically assigning different weights to gene-gene interactions, multi-head attention captures both global regulatory patterns and local dependencies, enhancing gene feature learning and supporting the construction of robust gene regulatory networks.

A scoring function *e*: $R^{n}\times R^{n}\to R$ computes a score for two genes share a common hyperedge $\left(h_{i},h_{j}\right)$, which indicates the importance of the expression profiles of two genes $v_{i}$ and $v_{j}$, which belong to the same hyperedge $e_{k}$:

(Equation 4) $$e\left(h_{i}^{\left(l\right)},h_{j}^{\left(l\right)}\right)=\text{LeakyReLU}\left(a^{T}\cdot \left[{Wh}_{i}^{\left(l\right)}\|{Wh}_{j}^{\left(l\right)}\right]\right)\text{,}$$

where $a\in R^{2n^{\prime }}$, $W\in R^{n^{\prime }\times n}$ are trainable parameters, and ‖ denotes vector concatenation. These attention scores are normalized across all hyperedges using softmax, and the attention function is defined as:

(Equation 5) $$\begin{array}{cc} \alpha_{ij} & ={\text{softmax}}_{j}\left(e\left(h_{i}^{\left(l\right)},h_{j}^{\left(l\right)}\right)\right)=\frac{\exp\left(e\left(h_{i}^{\left(l\right)},h_{j}^{\left(l\right)}\right)\right)}{\sum_{j^{\prime }\in e_{k}}\;\exp\left(e\left(h_{i}^{\left(l\right)},h_{j^{\prime }}^{\left(l\right)}\right)\right)}\text{,} \end{array}$$

We denote the coefficient matrix, whose entries are $\alpha_{ij}$, if $\left(h_{i}^{\left(l\right)},h_{j^{\prime }}^{\left(l\right)}\right)\in e_{k}$, and 0 otherwise. Then, GAT computes a weighted average of the transformed features of the neighbor nodes followed by a non-linearity *σ* as the new representation of $h_{i}$, using the normalized attention coefficients:

(Equation 6) $$h_{i}^{\left(l+1\right)}=\sigma \left(\sum_{j\in e_{k}}\alpha_{ij}\cdot \left(M\Omega M^{T}h_{j}^{\left(l\right)}\right)W\right)\text{,}$$

In layer (l+1), the representation of $h_{i}$ is denoted by $h_{i}^{\left(l+1\right)}$. The hyperedge weight matrix $\Omega \in R^{n\times n}$, is set as the identity matrix, due to the lack of prior knowledge regarding cell relationships. In this paper, we refer to (Equation 4), (Equation 5), (Equation 6) as the computation of each layer in an *L*-layer HyperG-VAE node encoder. We also leveraged the multi-head attention mechanism, akin to the strategy used in Vaswani et al.^68^ to stabilize the learning process of self-attention.

Through the message-passing layers, the input node features of $H^{V}$ could be represented as ${\tilde{Z}}^{V}$, two individual fully connected layers are then employed to estimate the means $\mu^{V}$ and variances $\sigma^{V}$ of $q\left(Z^{V}|H^{V};\theta^{V}\right)$:

(Equation 7) $$\mu^{V}={\tilde{Z}}^{V}W_{\mu }^{V}+b_{\mu }^{V}\text{,}$$

(Equation 8) $$\sigma^{V}={\tilde{Z}}^{V}W_{\sigma }^{V}+b_{\sigma }^{V}\text{,}$$

where $W_{\mu }^{V}$, $W_{\sigma }^{V}\in R^{d_{out}\times d}$, *d* is the dimensionality of the final node embedding $Z^{V}$, which is sampled by the following process:

(Equation 9) $$Z^{V}=\mu^{V}+\sigma^{V}⊙\epsilon \text{,}$$

where ϵ∼N(0,I) and is scaled element-wise by $\sigma^{V}$. The collective set of parameters, encapsulated within $\theta^{V}$, offers the posterior estimates for $q\left(Z^{V}|H^{V};\theta^{V}\right)$.

#### HyperG-VAE hyperedge encoder

Based on the Equation 1 and nonlinear version of the SEM proposed by,^80^ the encoder part of the SEM variational autoencoder could be represented as:

(Equation 10) $$Z=f_{2}\left(\left(I-A^{T}\right)f_{1}\left(H^{E}\right)\right)\text{,}$$

here, the functions $f_{1}$ and $f_{2}$, parameterized for potential non-linear transformations, adeptly act upon $H^{E}$ and Z, respectively.

Based on Equation 10, to encode the high-order semantics and complex relations represented in the form of hyperedges, a hyperedge encoder first conducts a non-linear feature transformation from the observed embedding $H^{E}$ into a common latent space ${\tilde{Z}}^{E}$, which is as follows:

(Equation 11) $${\tilde{Z}}^{E}=\left(I-A^{T}\right)f_{E}\left(H^{E}X^{V}W^{E}+b^{E}\right)\text{,}$$

While the gene expression profile is given by $h_{i}$, $X^{V}\in R^{m\times f}$ denotes the initial *f*-dimensional gene features matrix. Due to the absence of this detailed feature information in our dataset, $X^{V}$ is simplified as an identity matrix, I. $f_{E}$ stands for multilayer neural network, $W^{E}$ is the learnable weight matrices, and $b^{E}$ is bias.

Given the fused hyperedge embedding ${\tilde{Z}}^{E}$, two individual fully connected layers are then employed to estimate the means $\mu^{E}$ and variances $\sigma^{E}$ of $q\left(Z^{E}|H^{V};\theta^{E}\right)$:

(Equation 12) $$\mu^{E}={\tilde{Z}}^{E}W_{\mu }^{E}+b_{\mu }^{E}\text{,}$$

(Equation 13) $$\sigma^{E}={\tilde{Z}}^{E}W_{\sigma }^{E}+b_{\sigma }^{E}\text{,}$$

where $W_{\mu }^{E}$, $W_{\sigma }^{E}\in R^{d_{out}^{\prime }\times d^{\prime }}$, $d^{\prime }$ is the dimensionality of the $Z^{E}$, which is sampled by the following process:

(Equation 14) $$Z^{E}=\mu^{E}+\sigma^{E}⊙\epsilon \text{,}$$

where ϵ∼N(0,I) and is scaled element-wise by $\sigma^{E}$. The collective set of parameters, encapsulated within $\theta^{E}$, offers the posterior estimates for $q\left(Z^{E}|H^{V};\theta^{E}\right)$.

#### Generative model

In the decoding phase, the hypergraph is reconstructed utilizing the latent space representations, $Z^{V}$ and $Z^{E}$, acquired from the node and hyperedge encoders, respectively.

To keep the nonlinear SEM of the hyperedge encoder, we first reconstruct the representation of $H^{E}$, and we use the corresponding decoder of Equation 10:

(Equation 15) $$H^{E}=f_{4}\left({\left(I-A^{T}\right)}^{-1}f_{3}\left(Z\right)\right)\text{,}$$

In this work, we can represent the inner content of $f_{4}$ as:

(Equation 16) $${\tilde{Z}}^{E^{\prime }}={\left(I{-}^{AT}\right)}^{-1}f_{E^{\prime }}\left(Z^{E}W^{E^{\prime }}+b^{E^{\prime }}\right)\text{,}$$

where $W^{E^{\prime }}$ is the learnable weight matrices, and $b^{E^{\prime }}$ is bias. Correspondingly, we can get the estimated means $\mu^{E^{\prime }}$ and variances $\sigma^{E^{\prime }}$ based on $Z^{E}$:

(Equation 17) $$\mu^{E^{\prime }}=Z^{E}W_{\mu }^{E^{\prime }}+b_{\mu }^{E^{\prime }}\text{,}$$

(Equation 18) $$\sigma^{E^{\prime }}=Z^{E}W_{\sigma }^{E^{\prime }}+b_{\sigma }^{E^{\prime }}\text{,}$$

where $W_{\mu }^{E^{\prime }}$, $W_{\sigma }^{E^{\prime }}\in R^{d_{out}\times d}$, *d* is the dimensionality of the final hyperedge representation $Z^{E^{\prime }}$, which is sampled by the following process:

(Equation 19) $$Z^{E^{\prime }}=\mu^{E^{\prime }}+\sigma^{E^{\prime }}⊙\epsilon \text{,}$$

where ϵ∼N(0,I) and is scaled element-wise by $\sigma^{E^{\prime }}$.

Finally, the estimated hypergraph based on distributions $p\left(H^{V}|Z^{V},Z^{E};\lambda^{V}\right)$ is represented as:

(Equation 20) $${\tilde{H}}^{V}=Z^{V}⊙Z^{E^{\prime }}\text{.}$$

#### Hypergraph variational evidence lower bound

In the process of HyperG-VAE, latent node embeddings $Z^{V}$ and high-order relation embeddings $Z^{E}$ are first generated independently from a parameter-free prior distribution, typically a Gaussian. The observed data points $H^{V}$ are then generated conditionally, based on these latent embeddings, with each data point being conditioned on its corresponding latent node embedding $Z^{V}$ and high-order relation embeddings $Z^{E}$, parameterized by λ. The objective of HyperG-VAE is to optimize these parameters λ to maximize the log likelihood of the observed data. To derive a lower bound for the log likelihood, known as the Evidence Lower Bound (ELBO). HyperG-VAE leverages Jensen’s Inequality as follows:

(Equation 21) $$\begin{array}{cc} \log\;p\left(H^{V};\lambda \right) & =\log\;\int_{Z^{V}}\int_{Z^{E}}p\left(H^{V},Z^{V},Z^{E};\lambda \right)dZ^{V}dZ^{E}\\ & \geq E_{q}\left[\log\frac{p\left(H^{V},Z^{V},Z^{E};\lambda \right)}{q\left(Z^{V},Z^{E}|H^{V};\theta \right)}\right]\\ & :=L\left(H^{V};\theta ,\lambda \right)\text{,} \end{array}$$

where $q\left(Z^{V},Z^{E}|H^{V};\theta \right)$ is the variational posterior used to approximate the true posterior $p\left(Z^{V},Z^{E}|H^{V}\right)$, and θ is the parameter that we need to estimate in the learning phase. The Evidence Lower Bound (ELBO) on the marginal likelihood of $H^{V}$, denoted as $L\left(H^{V};\theta ,\lambda \right)$, is derived by applying the logarithmic product rule to the joint probability distribution, facilitating a tractable lower bound for model optimization:

(Equation 22) $$\begin{array}{cc} L\left(H^{V};\theta ,\lambda \right) & =E_{q}\left[\log\left(\frac{p\left(H^{V}|Z^{V},Z^{E};\lambda \right)p\left(Z^{V},Z^{E}\right)}{q\left(Z^{V},Z^{E}|H^{V};\theta \right)}\right)\right]\\ & =E_{q}\left[\log\;p\left(H^{V}|Z^{V},Z^{E};\lambda \right)\right]\\ & -KL\left(q\left(Z^{V}|H^{V};\theta^{V}\right)\|p\left(Z^{V}\right)\right)\\ & -KL\left(q\left(Z^{E}|H^{V};\theta^{E}\right)\|p\left(Z^{E}\right)\right)\text{,} \end{array}$$

In the variational autoencoder framework, specifically within the HyperG-VAE, the Kullback-Leibler (KL) divergence acts as a regularization factor. It aligns the variational distribution q(·|·;θ) with the prior distribution p(·), reinforcing the model’s adherence to initial assumptions. Concurrently, the expected log likelihood of reconstruction, expressed as E[logp(·|λ)], dictates the fidelity of data reconstruction from latent embeddings, which are shaped by the learned distribution. The parameter λ, crucial to this reconstruction, is optimized during the learning phase. This dual mechanism ensures that while the model is incentivized to replicate observed data accurately, it remains regularized by the prior, establishing a balance pivotal to the ELBO’s effectiveness in training variational models like HyperG-VAE.

$H^{V}$ and $H^{E}$ are transposed relations. To better tailor the learning process to specific objectives, weighting components within a loss function, as in Beta-VAE,^82^ offers nuanced control over regularization, fostering more interpretable and generalizable models. And we will get the ELBO used in HyperG-VAE as:

(Equation 23) $$\begin{array}{cc} L\left(H^{V};\theta ,\lambda \right) & =E_{q}\left[\log\;p\left(H^{V}|Z^{V},Z^{E};\lambda^{V}\right)\right]\\ & -\alpha KL\left(q\left(Z^{V}|H^{V};\theta^{V}\right)∥p\left(Z^{V}\right)\right)\\ & -\beta KL\left(q\left(Z^{E}|H^{E};\theta^{E}\right)∥p\left(Z^{E}\right)\right) \end{array}$$

#### Model setup

HyperG-VAE was devised to infer gene regulatory networks from scRNA-seq data without relying on cell type annotations. Before feeding into the model, the scRNA-seq expression data underwent log-transformation followed by Z-normalization to ensure optimal data representation. For the initialization of the gene interaction matrix, denoted as $\tilde{A}$, the matrix diagonal was set to zeros, while the other entries followed a Gaussian distribution $N\left(1/\left(m-1\right),\epsilon^{2}\right)$. Here, *m* represents the number of genes, and ϵ is a small value introduced to prevent entrapment in local optima.

We chose a two-step alternative optimization approach. The RMSprop algorithm^83^ was selected initially for tuning the weights within the HyperG-VAE layers over specific epochs. Then, in a separate phase, the weight matrix $\tilde{A}$, which plays a critical role in our architecture, was fine-tuned over another set of epochs, employing a differential learning rate strategy. This bifurcated approach not only fortified the model’s robustness but also ensured granular weight updates in both the matrix and the neural layers. We utilized the kaiming_uniform technique^84^ to initialize MLPs, crucially defining the initial conditions of our model. The gene (node) encoder, taking the constructed hypergraph as input, employs the Xavier uniform initialization^85^ for optimal training. During training, the model’s objective function was guided by a multi-faceted loss: a reconstruction component to maintain data fidelity, two KL divergences (sourced from both the node encoder and the hyperedge encoder) to ensure latent variable alignment with *a priori* distributions, and a penalty promoting sparsity in the adjacency matrix. This ensured both accuracy in reconstruction and interpretability in inferred gene interactions.

This holistic framework was crafted in Python and leaned heavily on the computational prowess of the PyTorch framework,^86^ complemented by scanpy^81^ for preliminary data handling. Key hyperparameters are selected based on a grid search strategy, more details could be checked in Table S1. More details of the structure introduction of HyperG-VAE can be found in the Supplementary.

#### GRN inference

Central to our model, HyperG-VAE, the gene regulatory network (GRN) is elucidated through the learned causal interaction matrix $\tilde{A}$. The absolute values within this matrix convey the potential links between genes, reflecting the probability of their interrelations. To enhance the biological interpretability of the predicted GRNs, we incorporate SCENIC,^41^ a method renowned for its ability to distill biologically meaningful gene regulations. SCENIC’s capability to identify cell-type-specific regulatory interactions complements the precision of HyperG-VAE, providing a deep learning-based approach that bridges the accuracy of causal gene interactions with the biological relevance of transcription factor (TF)-gene relationships.

HyperG-VAE first identifies high-confidence TF-gene pairs by modeling complex gene dependencies and direct regulatory links, precisely capturing causal relationships while accounting for cell heterogeneity and gene-specific regulatory modules. SCENIC is then applied to filter and refine these TF-gene pairs, further enhancing their biological relevance. By using motif enrichment analysis and transcription factor activity scoring, SCENIC identifies the key transcription factors that drive gene expression in specific cell types, ensuring that the resulting GRNs are biologically meaningful and contextually relevant. This cascading combination of HyperG-VAE and SCENIC enables the construction of robust, biologically grounded gene regulatory networks. Together, they offer a comprehensive view of gene regulation, not only uncovering the structure of the GRNs but also providing insights into their functional significance.

#### Gene set enrichment analysis (GSEA)

For the analysis of gene clusters, we employed the default settings of Metascape.^43^ Specifically, enrichment analysis for given gene lists encompassed pathway and process assessments using GO Biological Processes, GO Cellular Components, GO Molecular Functions, and DisGeNET ontologies. The entire genome served as the background for enrichment. Terms meeting stringent criteria: *p*-value ⟨ 0.01, minimum count of 3, and enrichment factor > 1.5 (ratio of observed to expected counts)were selected. Statistical rigor was maintained by employing cumulative hypergeometric distribution for *p*-value calculation, Banjamini-Hochberg procedure for q-value adjustment, and Kappa scores for hierarchical clustering. Clusters, defined by sub-trees with a similarity exceeding 0.3, were identified based on membership similarities. Each cluster is represented by its most statistically significant term. This comprehensive approach ensures robust and reliable insights into gene function and pathway associations.

#### Latent representation visualization and clustering

In both HyperG-VAE and the comparative methodologies, if the size of hidden embeddings exceeded 10, we commenced by extracting the foremost 10 principal components (PCs) via principal component analysis. Subsequently, a cell neighborhood graph was computed, setting the “n_neighbors” parameter to 30. Visualization of dataset results was then performed in a two-dimensional space using the default parameters of the UMAP algorithm. For cell clustering, the Louvain algorithm was employed, and the “resolution” parameter was fine-tuned using a binary search to yield a cluster count consistent with cell-type annotations.

#### Datasets used for GRN evaluation

We evaluate the performance on GRN inference of HyperG-VAE based on the setting of BEELINE framework.^27^ Our evaluation encompassed seven scRNA-seq datasets. This includes two cell lines from human, human embryonic stem cells (hESC)^87^ and human mature hepatocytes (hHEP).^88^ Additionally, five mouse cell lines are studied here: mouse dendritic cells (mDC),^89^ mouse embryonic stem cells (mESC),^37^ mouse hematopoietic stem cells with erythroid-lineage (mHSC-E),^90^ mouse hematopoietic stem cells with granulocyte-monocyte-lineage (mHSC-GM)^90^ and mouse hematopoietic stem cells with lymphoid-lineage (mHSC-L).^90^ Furthermore, the EPR and AUPRC the GRN performance based on four kinds of groundstruth: STRING,^28^ Non-specific ChIP-seq,^29^^,^^30^^,^^31^ Cell-type-specific ChIP-seq,^32^^,^^33^^,^^34^ and loss-/gain-of-function (LOF/GOF) groundtruth network.^34^ Following the guidelines outlined by Pratapa et al.,^27^ our dataset-specific analysis emphasized the most variable transcription factors and considered the top N most-varying genes, with N being 500 and 1,000. We meticulously adhered to the raw data preprocessing steps detailed in their work and, for evaluation, disregarded any edges that did not originate from TFs. More details can be found in Table S2 and S3.

#### scRNA-seq datasets of bone marrow developmental B cells

We assess the overarching capability of HyperG-VAE in modeling gene regulatory networks pivotal to B cell development and transformation based on previously published bone marrow developmental B cells datasets.^38^ The raw sequencing data in this study were processed using the CellRanger pipeline (version 3.1.0, 10X Genomics), where the “mkfastq” function demultiplexed three Illumina libraries (mRNA transcript expression (RNA), mouse-specific hashtag oligos (HTO), and cell surface marker levels using antibody-derived tags (ADT)) and “count” aligned reads to the mouse genome (mm10) to generate count tables. Analysis was carried out in R using the Seurat package,^91^ involving filtering of the RNA dataset to include only GEMs expressing more than 300 genes and excluding those with high mitochondrial RNA levels. Normalization was performed using a centered-log ratio method. Doublets were identified in GEMs using both DoubletFinder and HTODemux methods; however, due to discrepancies in classification and challenges with DoubletFinder in identifying similar doublets, subsequent analyses relied solely on HTODemux classifications. GEMs identified as multiplets or negative were removed, leaving a refined dataset of wildtype (WT) singlets, which expressed a median of 1409 genes with 3548 counts. These WT singlets then underwent a transformation process using Seurat’s “SCTransform” function, factoring in the percentage of mitochondrial expression, to prepare a high-quality, normalized dataset for further study.

#### Datasets used for cellular heterogeneity study

We assessed the efficacy of HyperG-VAE by applying it to three pertinent scRNA-seq datasets: an Alzheimer’s disease (AD) study,^57^ a colorectal cancer investigation,^58^ and the renowned mouse brain dataset, often referred to as the Zeisel dataset.^59^ HyperG-VAE processes raw scRNA-seq gene expression profiles directly. The initial phase of data preprocessing involves rigorous data filtering and quality control. Considering the significant dropout rates characteristic of scRNA-seq expression datasets, only genes with non-zero expression in over 1% of cells and cells with non-zero expression in more than 1% of genes are retained. Subsequently, genes are ranked based on their standard deviation, and the top 2,000 genes in terms of variance are selected for further analysis. More details can be found in Table S4.

#### SCENIC and Chip-Atlas setting

In our approach to further filter reliable gene regulatory networks (GRN) from single-cell RNA-sequencing data, we integrated HyperG-VAE with SCENIC, focusing on discerning crucial gene co-expression modules. Specifically, only the top 0.5% of gene pairs predicted by HyperVAE, based on their co-expression significance, are channeled into SCENIC for rigorous regulon analysis. Using the MusMusculus genome reference, our model evaluates regulatory regions defined as 500 bp upstream, 5-kb, and 10-kb centered around each gene’s transcription start site (TSS), collectively referred to as gene-motif rankings. The analysis adopts criteria for GRN derivation of SCENIC: a feature AUC (default: 0.05), gene rank threshold (default: 5,000), and a normalized enrichment score (NES) threshold (default: 3.0).

To validate the predicted regulons, we cross-verified our computational results with publicly available ChIP-seq datasets.^33^ Following the foundational settings of SCENIC, we specifically tailored the study to the M. musculus (mm9) genome. Furthermore, in our evaluation approach, we incorporated multiple transcription start sites (TSS) ranges, including 1k, 5k, and 10k, to ensure a comprehensive understanding of gene expression.

### Quantification and statistical analysis

To evaluate the performance of HyperG-VAE, we employed multiple statistical and quantitative analyses. These methods assess the accuracy, robustness, and reliability of the inferred GRNs, clustering assignments, and motif enrichment results.

#### GRN inference evaluation metrics

To assess the accuracy of the inferred GRNs, we compared the predicted networks to ground-truth GRNs using the following metrics.

(1) EPR is defined as the odds ratio of the true positives among the top K predicted edges between the model and the random predictions where K denotes the number of edges in ground-truth GRN.
(2) AUPRC ratio is defined as the odds ratio of the area under the precision-recall curve (AUPRC) between the model and the random predictions.
(3) The Overlap coefficient is a similarity measure related to the Jaccard Similarity, but whereas the Jaccard Similarity considers both the intersection and union of two sets, the Overlap Coefficient only considers the intersection relative to the smaller set. It’s used to quantify the overlap between two sets. Given two sets, *A* and *B*, the Overlap Coefficient *O* is defined as:

$$O\left(A,B\right)=\frac{\left|A\cap B\right|}{\min\left(\left|A\right|,\left|B\right|\right)}$$

The value of the Overlap Coefficient lies between 0 and 1: A value of 1 indicates that the sets are identical, and 0 indicates that the sets have no elements in common.

#### Clustering performance metrics

To evaluate cell clustering quality, we compared the inferred clusters with known cell populations using four clustering similarity metrics.

(1) NMI (Normalized Mutual Information) quantifies the mutual dependence between two clustering assignments, offering a value between 0 (completely independent assignments) and 1 (identical assignments).
(2) ARI (Adjusted Rand Index) is an adjusted variant of the Rand Index that gauges clustering similarity while accounting for random agreement. Its values range from −1 (perfect disagreement) to 1 (perfect agreement), with 0 indicating random agreement.
(3) HOM (Homogeneity) evaluates whether each cluster comprises solely members of a single class. It ranges from 0 (poor homogeneity) to 1 (perfect homogeneity).
(4) COM (Completeness) assesses if all members of a given class are confined to the same cluster, with scores spanning from 0 (low completeness) to 1 (perfect completeness).

#### Motif enrichment analysis

To validate the biological relevance of the inferred GRNs, we performed motif enrichment analysis using the Normalized Enrichment Score (NES).

(1) The Normalized Enrichment Score (NES) quantifies the enrichment of a given motif at the top of a ranking compared to motifs generated by chance. Mathematically, NES is defined as:

$$\text{NES}=\frac{{\text{AUC}}_{\text{motif}}-\text{mean}\left({\text{AUC}}_{\text{all}\;\text{motifs}}\right)}{s.d.\left({\text{AUC}}_{\text{all}\;\text{moti}}\right)}$$

(1) where ${\text{AUC}}_{\text{motif}}$ represents the Area Under the Curve for the top 0.5% of the ranked motifs for the gene of interest, and the mean and standard deviation are calculated across the AUCs of all motifs in the dataset. A higher NES indicates a more significant enrichment of the motif in the given context.

## Supplemental information

Document S1. Figures S1–S5 and Tables S1–S6

Document S2. Article plus supplemental information
