# Supplementary material for: Inferring gene regulatory networks by hypergraph generative model
Source: Cell Rep Methods. 2025 Apr 11;5(4):101026. doi: 10.1016/j.crmeth.2025.101026 (PMC12256954; doi:10.1016/j.crmeth.2025.101026)
Supplement: Document S1. Figures S1–S5 and Tables S1–S6 [file mmc1.pdf]

Cell Reports Methods, Volume *5*

Supplemental information

Inferring gene regulatory networks by hypergraph generative model

Guangxin Su, Hanchen Wang, Ying Zhang, Marc R. Wilkins, Pablo F. Canete, Di Yu, Yang Yang, and Wenjie Zhang

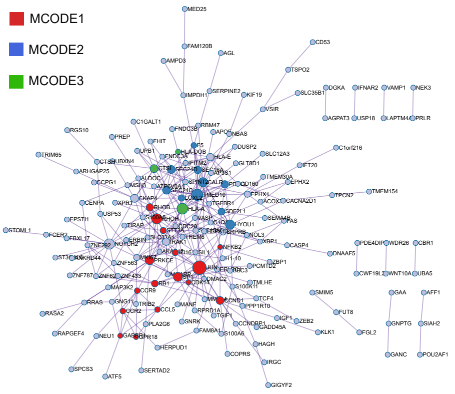

(a) The MCODE network identified for Plasma B gene lists.

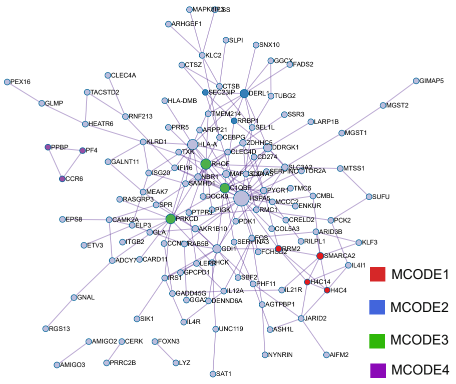

(b) The MCODE network identified for Kappapre B gene lists.

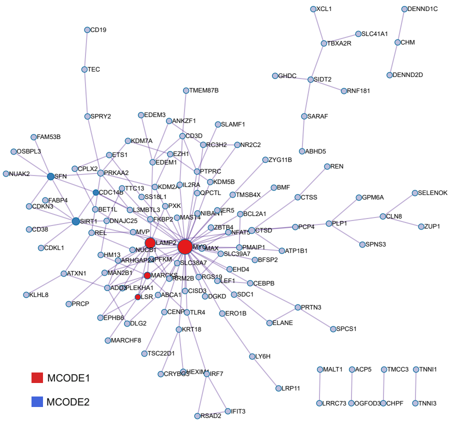

(c) The MCODE network identified for PreBCRi B gene lists.

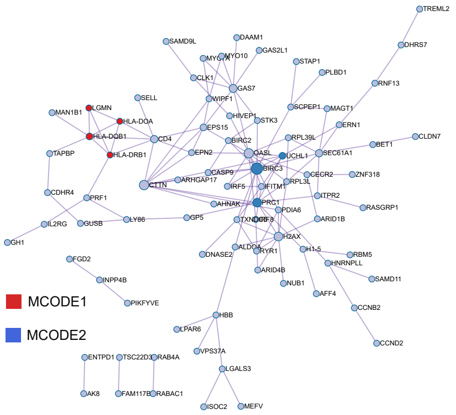

(d) The MCODE network identified for Immature B gene lists.

Figure S1: **The MCODE network identified for gene lists of different B cell states. Related to Figure 4.**

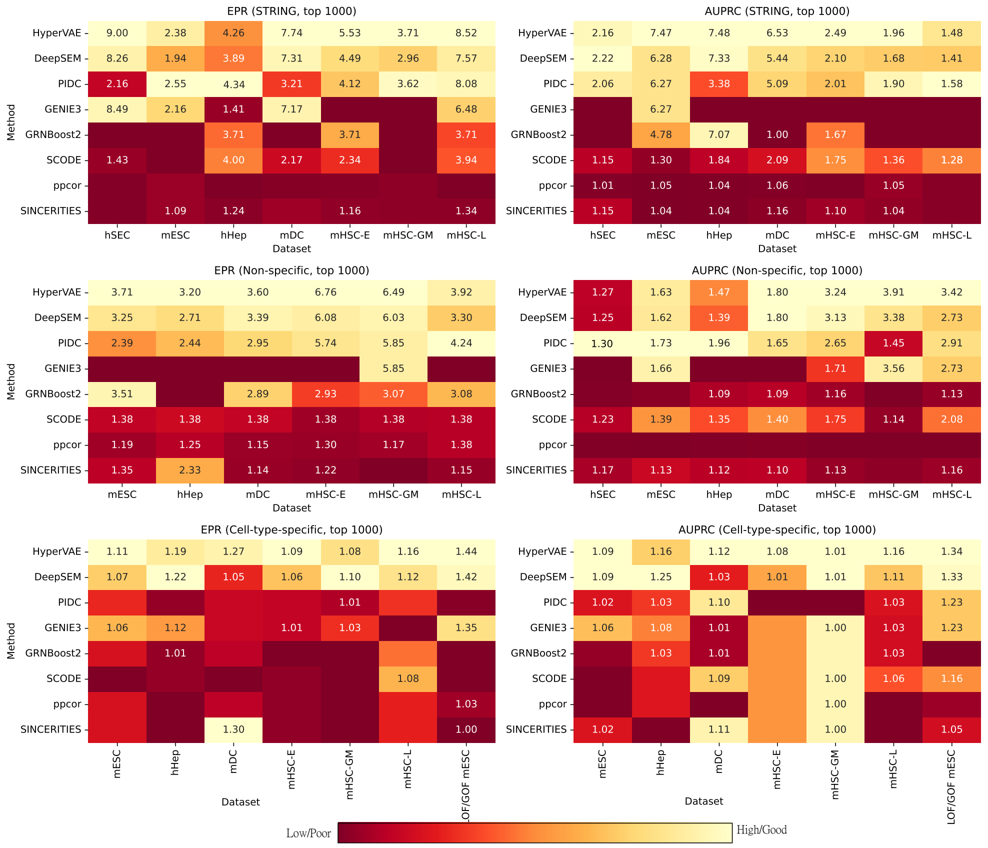

Figure S2: **Benchmarks of different GRN inference methods on experimental single-cell RNA-seq datasets by EPR and AUPRC scores. Related to Figure 2.** The performance of HyperG-VAE is contrasted against seven alternative algorithms across seven datasets. Each dataset comprises all significantly varying transcription factors (TFs) and the 1000 most varying genes. These evaluations are based on four distinct ground-truth benchmarks: Non-specific ChIP-seq, STRING, Cell-type-specific ChIP-seq, and LOF/GOF. For each figure pair, the left panel depicts the median EPR results, while the right panel shows the median AUPRC outcomes. Results inferior to random predictions are excluded from the visualizations for clarity. The color scale in each dataset is normalized between 0 and 1 using a min–max scaling approach. EPR is defined as the odds ratio of true positives among the top K predicted edges, where K represents the number of edges in the ground-truth GRN, compared to random predictions. Similarly, the AUPRC ratio reflects the odds ratio of the AUPRC value between the model and random predictions.

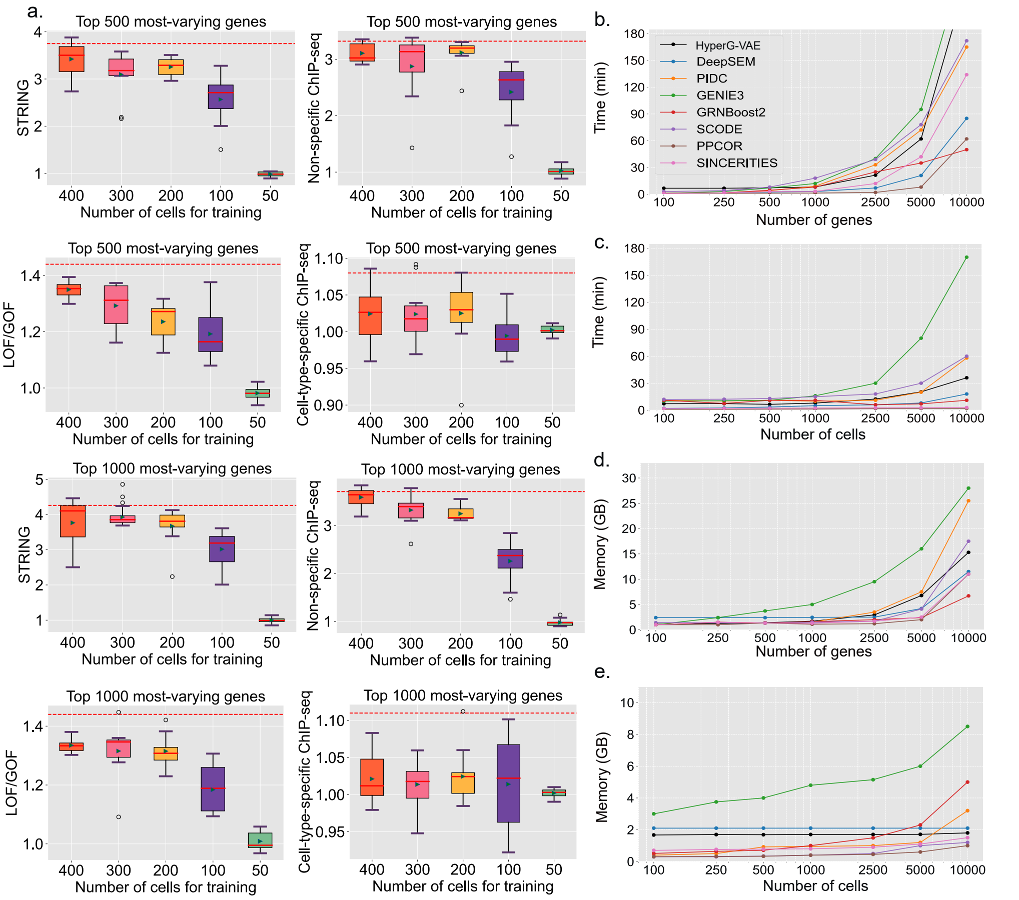

Figure S3: **The EPR performance of HyperG-VAE with the limited number of training cells. And, running time and memory cost of different methods on the simulated datasets. Related to Figure 2. a,** mESC datasets composed of all significantly varying TFs and the 500/1000 most-varying genes are evaluated based on four unique groundtruth benchmarks: STRING, Non-specific ChIP-seq, Cell-type-specific ChIP-seq, and LOF/GOF. The visualization captures the median (represented by the internal line), the interquartile range (shown by the box), and the whiskers (which stretch to 1.5 times the interquartile range). Different colored boxes correspond to distinct training cell numbers, while the green markers within the boxes signify the mean values. Notably, the red dashed line represents the median EPR value across all cell counts. **b,** Running time of training HyperG-VAE and other GRN inference methods on a simulated dataset with 1000 cells when the number of genes for each cell increased. **c,** Running time for training HyperG-VAE and other GRN inference methods on a simulated dataset with 1000 genes for each cell when the number of cells increased. **d,** Memory cost of training HyperG-VAE and other embedding methods on a simulation dataset with 1000 cells when the number of genes for each cell increased. **e,** Memory cost of training HyperG-VAE and other embedding methods on a simulation dataset with 1000 genes for each cell when the number of cells increased.

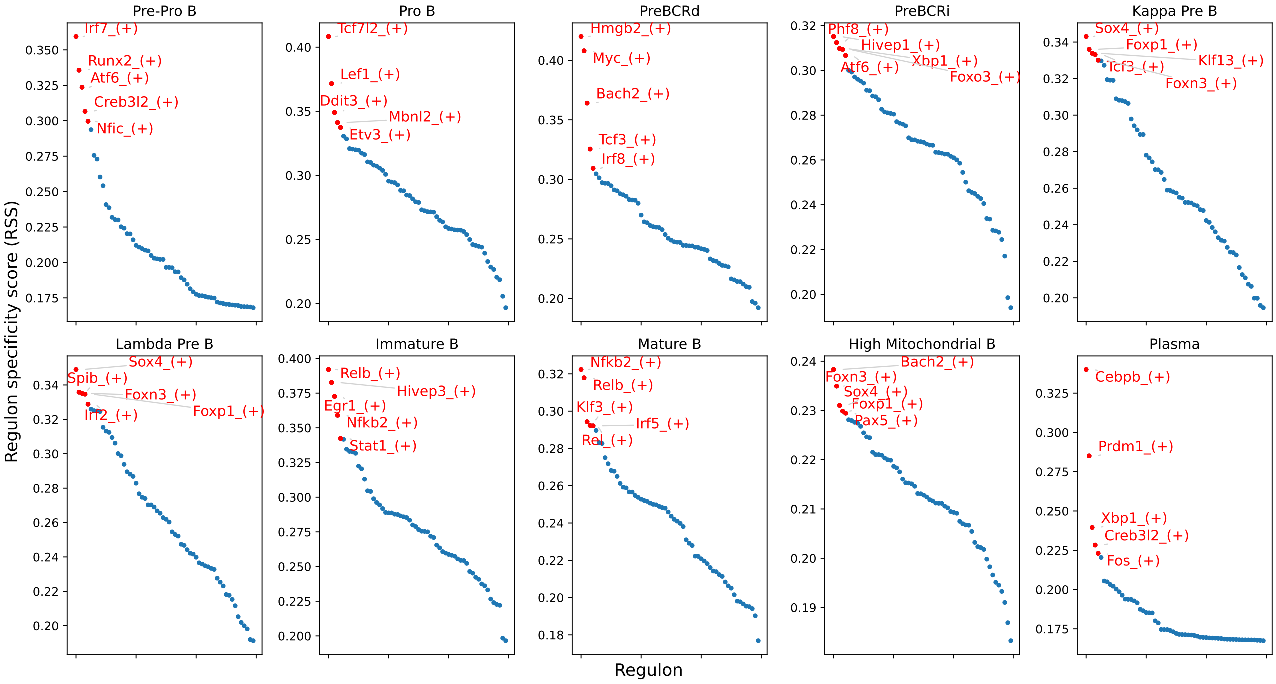

Figure S4: Regulon specificity score for each bone marrow B cell state. Related to Figure 3. The top five regulons in each cell type are highlighted in red and labeled on the plot. The specificity score is shown on the y-axis.

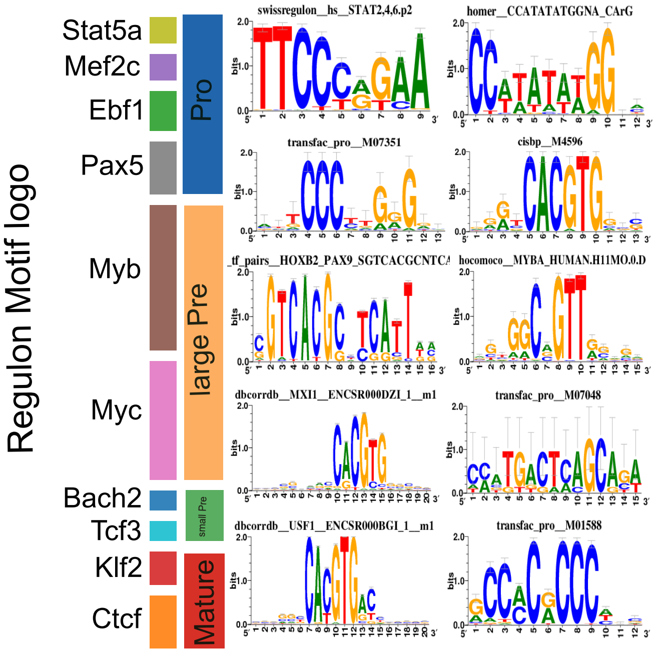

Figure S5: The motif of significant TFs along the four principal stages of Section HyperG-VAE constructs the cell-type-specific GRN on B cell development in bone marrow. Related to Figure 5.

Hyperparamters	Values
α	1, 10
β	0.1, 0.2
ω	0.1
Learning rate	0.001, 0.01, 0.0001
Weight decay	0.1
Batchsize	64, 128, 256
Dropout rate	0, 0.5
# of heads	6, 8
# of training epochs	70

Table S1: **Summary of tuned hyper-parameters. Related to Figure 2-6.**

Ground Truth	Cell type	Source	Download link
**STRING**	mESC, mDC, mHSC-E, mHSC-GM, mHSC-L, hESC, hHep	STRING [1]	https://string-db.org/
**Cell-type Non specific ChIP-seq**	mESC, mDC, mHSC-E, mHSC-GM, mHSC-L	TRRUST [2] RegNetwork [3]	https://www.grnpedia.org/trrust/ https://www.regnetworkweb.org
**Cell-type Non specific ChIP-seq**	hESC, hHep	TRRUST [2] RegNetwork [3] DoRothEA [4]	https://www.grnpedia.org/trrust/ https://www.regnetworkweb.org https://saezlab.github.io/dorothea/index.html
**Cell-type specific ChIP-seq**	mHSC-E, mHSC-GM, mHSC-L, mDC	ChIP-Atlas [5]	https://chip-atlas.org/peak_browser
**Cell-type specific ChIP-seq**	mESC	ChIP-Atlas [5] ESCAPE [6]	https://chip-atlas.org/peak_browser http//www.maayanlab.net/ESCAPE/download.php
**Cell-type specific ChIP-seq**	hESC, hHep	ChIP-Atlas [5] ChEA [7]	https://chip-atlas.org/peak_browser https://maayanlab.cloud/Harmonizome/dataset/CHEA+Transcription+Factor+Targets
**lof/gof**	mESC	ESCAPE [6]	http//www.maayanlab.net/ESCAPE/download.php
**scATAC-seq**	Mouse cortex	Fang et al. [8]	https://www.ncbi.nlm.nih.gov/geo/query/acc.cgi?acc=GSE126724
**snmC-seq (DMR)**	Mouse cortex	Luo et al. [9]	https://www.ncbi.nlm.nih.gov/geo/query/acc.cgi?acc=GSEGSE97179 Supplmentary Tables in Luo et al.

Table S2: **Summary of ground truth GRN networks used in the GRN predictions. Related to Figure 2.**

Dataset	# of cells	# of genes	GEO
Human embryonic stem cells (hESC) [10]	759	17735	GSE75748
Human mature hepatocytes (hHep) [11]	426	11515	GSE81252
Mouse dendritic cells (mDC) [12]	384	7371	GSE48968
Mouse embryonic stem cells (mESC) [13]	422	18385	GSE98664
Erythroid lineages mouse hematopoietic stem cells (mHSC-E) [14]	1072	4762	GSE81682
Lymphoid lineages mouse hematopoietic stem cells (mHSC-L) [14]	848	4762	GSE81682
Granulocyte-macrophage lineages mouse hematopoietic stem cells (mHSC-GM) [3]	890	4762	GSE81682
Mouse cortex VISp (L2/3 IT, L4, L5 IT, L5 PT, L6 IT, L6 CT) [15]	6456	31301	GSE115746

Table S3: Summary of scRNA-seq datasets used in GRN prediction. Related to Figures 3, 4, and 5.

Dataset	# of cells/nuclei	# of filtered genes	# of cell types	Download link
**B cell development data in bone marrow** [16]	3902	14003	10	https://www.ncbi.nlm.nih.gov/geo/query/acc.cgi?acc=GSE168158
**Zeisel** [17]	3005	2000	7	http://www.ncbi.nlm.nih. gov/geo/query/acc.cgi?a cc= GSE60361
**Li** [18]	561	2000	9	https://www.ncbi.nlm.nih .gov/geo/query/acc.cgi ? acc=GSE81861
**AD** [19]	13214	2000	8	https://www.ncbi.nlm.nih.gov/geo/query/acc.cgi?acc=GSE138852

Table S4: Summary of datasets used in embedding visualization and clustering. Related to Figure 6.

GO Category	Description	Log10(P)	Count	%InGO	Log10(q)
Plasma					
GO:0034976	Response to ER Stress	-10.96	17	6.56%	-6.61
GO:0001775	Cell Activation	-9.12	26	10.04%	-5.08
hsa05169	Epstein-Barr Virus Infection	-8.58	14	5.41%	-4.71
GO:0030335	Positive Regulation of Cell Migration	-7.60	21	8.11%	-3.95
GO:0031347	Regulation of Defense Response	-7.60	25	9.65%	-3.95
GO:0051251	Positive Regulation of Lymphocyte Activation	-6.76	15	5.79%	-3.41
hsa04142	Lysosome	-6.65	10	3.86%	-3.35
GO:2001233	Regulation of Apoptotic Signaling Pathway	-6.60	16	6.18%	-3.33
R-HSA-6798695	Neutrophil Degranulation	-5.99	17	6.56%	-2.91
R-HSA-446203	Asparagine N-Linked Glycosylation	-5.59	13	5.02%	-2.68
GO:0031638	Zymogen Activation	-5.47	6	2.32%	-2.59
hsa05224	Breast Cancer	-5.28	9	3.47%	-2.46
GO:0010942	Positive Regulation of Cell Death	-5.22	18	6.95%	-2.43
GO:0002274	Myeloid Leukocyte Activation	-5.13	9	3.47%	-2.39
WP5322	CKAP4 Signaling Pathway Map	-5.13	8	3.09%	-2.39
hsa04010	MAPK Signaling Pathway	-5.00	12	4.63%	-2.30
GO:0071345	Cellular Response to Cytokine Stimulus	-4.70	19	7.34%	-2.08
GO:0030097	Hemopoiesis	-4.64	18	6.95%	-2.04
WP3888	VEGFA-VEGFR2 Signaling	-4.61	14	5.40%	-2.03
hsa05142	Chagas Disease	-4.54	7	2.70%	-1.99
Kappa Pre B					
GO:0071345	"Cellular Response to Cytokine Stimulus"	-7.90	21	10.77%	-3.55
GO:0045321	"Leukocyte Activation"	-7.17	18	9.23%	-3.22
R-HSA-6798695	"Neutrophil Degranulation"	-6.97	16	8.21%	-3.22
GO:0050778	"Positive Regulation of Immune Response"	-6.87	18	9.23%	-3.22
WP5115	"Network Map of SARS-CoV-2 Signaling Pathway"	-6.71	11	5.64%	-3.19
GO:0002449	"Lymphocyte Mediated Immunity"	-6.67	10	5.13%	-3.19
GO:0002831	"Regulation of Response to Biotic Stimulus"	-4.83	13	6.67%	-1.91
GO:0050730	"Regulation of Peptidyl-Tyrosine Phosphorylation"	-4.40	9	4.62%	-1.66
R-HSA-1280218	"Adaptive Immune System"	-4.37	16	8.21%	-1.63
GO:0007264	"Small GTPase Mediated Signal Transduction"	-4.34	9	4.62%	-1.63
GO:0009617	"Response to Bacterium"	-4.25	15	7.69%	-1.60
WP4313	"Ferroptosis"	-4.19	5	2.56%	-1.57
R-HSA-1280215	"Cytokine Signaling in Immune System"	-4.06	15	7.69%	-1.47
M5885	"NABA MATRISOME ASSOCIATED"	-3.93	15	7.69%	-1.36
hsa04612	"Antigen Processing and Presentation"	-3.82	5	2.56%	-1.28
hsa05200	"Pathways in Cancer"	-3.73	12	6.15%	-1.22
GO:0044242	"Cellular Lipid Catabolic Process"	-3.57	7	3.59%	-1.08
GO:0045653	"Negative Regulation of Megakaryocyte Differentiation"	-3.56	3	1.54%	-1.07
WP23	"B Cell Receptor Signaling Pathway"	-3.37	5	2.56%	-0.93
R-HSA-112040	"G-Protein Mediated Events"	-3.32	4	2.05%	-0.89

Table S5: **Pathway and Process Enrichment Analysis of Plasma and Kappa Pre B. Related to Figure 4.** Top 20 clusters with their representative enriched terms (one per cluster). "Count" is the number of genes in the user-provided lists with membership in the given ontology term. "%InGO" is the percentage of all of the user-provided genes that are found in the given ontology term (only input genes with at least one ontology term annotation are included in the calculation). "Log10(P)" is the p-value in log base 10. "Log10(q)" is the multi-test adjusted p-value in log base 10.

GO Category	Description	Log10(P)	Count	%InGO	Log10(q)
PreBCRi B					
GO:0046651	"Lymphocyte Proliferation"	-9.28	11	5.61%	-5.08
GO:0001775	"Cell Activation"	-8.72	22	11.22%	-4.90
WP23	"B Cell Receptor Signaling Pathway"	-7.83	9	4.59%	-4.44
GO:0006954	"Inflammatory Response"	-5.37	15	7.65%	-2.36
GO:0071345	"Cellular Response to Cytokine Stimulus"	-5.21	17	8.67%	-2.25
GO:1904064	"Positive Regulation of Cation Transmembrane Transport"	-5.15	8	4.08%	-2.23
GO:0019725	"Cellular Homeostasis"	-5.10	16	8.16%	-2.23
GO:0030163	"Protein Catabolic Process"	-4.93	17	8.67%	-2.07
WP2203	"Thymic Stromal Lymphopoietin (TSLP) Signaling Pathway"	-4.87	5	2.55%	-2.04
R-HSA-6798695	"Neutrophil Degranulation"	-4.77	13	6.63%	-1.99
GO:0031347	"Regulation of Defense Response"	-4.75	17	8.67%	-1.99
M195	"PID CMYB PATHWAY"	-4.74	6	3.06%	-1.98
GO:0033673	"Negative Regulation of Kinase Activity"	-4.67	9	4.59%	-1.95
GO:1902532	"Negative Regulation of Intracellular Signal Transduction"	-4.67	14	7.14%	-1.95
GO:0042100	"B Cell Proliferation"	-4.62	5	2.55%	-1.93
M145	"PID P53 DOWNSTREAM PATHWAY"	-4.49	7	3.57%	-1.87
GO:0046631	"Alpha-Beta T Cell Activation"	-4.23	6	3.06%	-1.71
GO:0006909	"Phagocytosis"	-4.09	7	3.57%	-1.63
GO:0051345	"Positive Regulation of Hydrolase Activity"	-4.01	13	6.63%	-1.57
GO:0043462	"Regulation of ATP-Dependent Activity"	-3.94	5	2.55%	-1.52
Immature B					
GO:0019886	"Antigen Processing and Presentation of Exogenous Peptide Antigen via MHC Class II"	-6.25	5	3.16%	-2.20
M7997	"SA Caspase Cascade"	-5.58	4	2.53%	-2.15
GO:0030036	"Actin Cytoskeleton Organization"	-4.57	12	7.59%	-1.52
WP707	"DNA Damage Response"	-4.50	5	3.16%	-1.49
GO:0002831	"Regulation of Response to Biotic Stimulus"	-4.35	11	6.96%	-1.38
WP5218	"Extrafollicular and Follicular B Cell Activation by SARS-CoV-2"	-4.33	5	3.16%	-1.38
hsa04144	"Endocytosis"	-4.26	8	5.06%	-1.37
R-HSA-877300	"Interferon Gamma Signaling"	-3.90	5	3.16%	-1.16
GO:0071900	"Regulation of Protein Serine/Threonine Kinase Activity"	-3.87	9	5.70%	-1.15
WP5115	"Network Map of SARS-CoV-2 Signaling Pathway"	-3.81	7	4.43%	-1.12
WP3646	"Hepatitis C and Hepatocellular Carcinoma"	-3.67	4	2.53%	-1.09
hsa04141	"Protein Processing in Endoplasmic Reticulum"	-3.54	6	3.80%	-0.99
GO:0031341	"Regulation of Cell Killing"	-3.52	5	3.16%	-0.98
M234	"PID IL2 STAT5 Pathway"	-3.29	3	1.90%	-0.82
GO:0043087	"Regulation of GTPase Activity"	-3.16	8	5.06%	-0.75
GO:0046649	"Lymphocyte Activation"	-3.06	9	5.70%	-0.68
M195	"PID CMYB Pathway"	-3.00	4	2.53%	-0.63
R-HSA-6798695	"Neutrophil Degranulation"	-3.00	9	5.70%	-0.63
GO:0009617	"Response to Bacterium"	-2.91	11	6.96%	-0.56
GO:0097190	"Apoptotic Signaling Pathway"	-2.90	7	4.43%	-0.55

Table S6: **Pathway and Process Enrichment Analysis of PreBCRi B and Immature B. Related to Figure 4.** Top 20 clusters with their representative enriched terms (one per cluster). "Count" is the number of genes in the user-provided lists with membership in the given ontology term. "%InGO" is the percentage of all of the user-provided genes that are found in the given ontology term (only input genes with at least one ontology term annotation are included in the calculation). "Log10(P)" is the p-value in log base 10. "Log10(q)" is the multi-test adjusted p-value in log base 10.

References

[1] Szklarczyk, D. *et al.* String v11: protein–protein association networks with increased coverage, supporting functional discovery in genome-wide experimental datasets. *Nucleic acids research* **47**, D607–D613 (2019).

[2] Han, H. *et al.* Trrust: a reference database of human transcriptional regulatory interactions. *Scientific reports* **5**, 11432 (2015).

[3] Liu, Z.-P., Wu, C., Miao, H. & Wu, H. Regnetwork: an integrated database of transcriptional and post-transcriptional regulatory networks in human and mouse. *Database* **2015**, bav095 (2015).

[4] Garcia-Alonso, L., Holland, C. H., Ibrahim, M. M., Turei, D. & Saez-Rodriguez, J. Benchmark and integration of resources for the estimation of human transcription factor activities. *Genome research* **29**, 1363–1375 (2019).

[5] Oki, S. *et al.* Ch ip-atlas: a data-mining suite powered by full integration of public ch ip-seq data. *EMBO reports* **19**, e46255 (2018).

[6] Xu, H. *et al.* Escape: database for integrating high-content published data collected from human and mouse embryonic stem cells. *Database* **2013**, bat045 (2013).

[7] Lachmann, A. *et al.* Chea: transcription factor regulation inferred from integrating genome-wide chip-x experiments. *Bioinformatics* **26**, 2438–2444 (2010).

[8] Fang, R. *et al.* Comprehensive analysis of single cell atac-seq data with snapatac. *Nature communications* **12**, 1337 (2021).

[9] Luo, C. *et al.* Single-cell methylomes identify neuronal subtypes and regulatory elements in mammalian cortex. *Science* **357**, 600–604 (2017).

[10] Chu, L.-F. *et al.* Single-cell rna-seq reveals novel regulators of human embryonic stem cell differentiation to definitive endoderm. *Genome biology* **17**, 1–20 (2016).

[11] Camp, J. G. *et al.* Multilineage communication regulates human liver bud development from pluripotency. *Nature* **546**, 533–538 (2017).

[12] Shalek, A. K. *et al.* Single-cell rna-seq reveals dynamic paracrine control of cellular variation. *Nature* **510**, 363–369 (2014).

[13] Hayashi, T. *et al.* Single-cell full-length total rna sequencing uncovers dynamics of recursive splicing and enhancer rnas. *Nature communications* **9**, 619 (2018).

[14] Nestorowa, S. *et al.* A single-cell resolution map of mouse hematopoietic stem and progenitor cell differentiation. *Blood, The Journal of the American Society of Hematology* **128**, e20–e31 (2016).

[15] Tasic, B. *et al.* Shared and distinct transcriptomic cell types across neocortical areas. *Nature* **563**, 72–78 (2018).

[16] Lee, R. D. *et al.* Single-cell analysis identifies dynamic gene expression networks that govern b cell development and transformation. *Nature communications* **12**, 6843 (2021).

[17] Zeisel, A. *et al.* Cell types in the mouse cortex and hippocampus revealed by single-cell rna-seq. *Science* **347**, 1138–1142 (2015).

[18] Li, H. *et al.* Reference component analysis of single-cell transcriptomes elucidates cellular heterogeneity in human colorectal tumors. *Nature genetics* **49**, 708–718 (2017).

[19] Grubman, A. *et al.* A single-cell atlas of entorhinal cortex from individuals with alzheimer's disease reveals cell-type-specific gene expression regulation. *Nature neuroscience* **22**, 2087–2097 (2019).
